# Tobacco treatment incorporating contingency management, nicotine replacement therapy, and behavioral counseling for pregnant women who use substances: a feasibility trial

**DOI:** 10.3389/fpsyt.2023.1207955

**Published:** 2023-08-16

**Authors:** Melissa A. Jackson, Amanda L. Brown, Amanda L. Baker, Billie Bonevski, Paul Haber, Yvonne Bonomo, Julie Blandthorn, John Attia, Natasha Perry, Daniel Barker, Gillian S. Gould, Adrian J. Dunlop

**Affiliations:** ^1^Hunter New England Health Local Health District, Newcastle, NSW, Australia; ^2^School of Medicine and Public Health, University of Newcastle, Callaghan, NSW, Australia; ^3^Hunter Medical Research Institute, New Lambton Heights, NSW, Australia; ^4^Drug and Alcohol Clinical Research Improvement Network, St. Leonards, NSW, Australia; ^5^College of Medicine and Public Health, Flinders University, Bedford Park, SA, Australia; ^6^Edith Collins Centre, Sydney Local Health District, Camperdown, NSW, Australia; ^7^Sydney Medical School, University of Sydney, Sydney, NSW, Australia; ^8^Department of Medicine, University of Melbourne, Parkville, VIC, Australia; ^9^Women's Alcohol and Drug Service, The Royal Women's Hospital, Parkville, VIC, Australia; ^10^Faculty of Health, Southern Cross University, Coffs Harbour, NSW, Australia

**Keywords:** tobacco treatment, smoking cessation, substance use disorders, pregnancy, contingency management, counseling, nicotine replacement therapy

## Abstract

**Introduction:**

Most pregnant women with substance use problems smoke, and few will quit during their pregnancy. Tobacco treatment is often overlooked, with the focus usually placed on other substance use. Additionally, few targeted effective treatments for this group exist. To address this, the feasibility of an intensive tobacco treatment incorporating contingency management (CM) that featured non–face-to-face delivery was examined.

**Methods:**

A single-arm pre-post design feasibility trial was conducted in three antenatal services that support women who use substances in metropolitan Australia. Participants were over the age of 15, had <33-week gestation, and smoked tobacco daily. They received financial incentives for daily carbon monoxide-verified smoking abstinence or reduction through an internet-based CM programme, nicotine replacement therapy (NRT) posted to women and partners or household members who smoked and telephone-delivered behavioral counseling from study enrolment to birth.

**Results:**

Of the 101 referrals, 46 women (46%) consented. The mean (SD) age was 31(±6) years, and the gestation period was 22(±6) weeks. Nineteen (41%) of those enrolled were retained for 12-week postpartum. Of 46 women, 32 (70%) utilized CM; 32 (70%) used NRT for ≥2 weeks; 23 (50%) attended ≥1 counseling session; and 15 (22%) received NRT for partners/household members. Fifteen (33%) were verified abstinent from tobacco at delivery after a median (IQR) period of abstinence of 65(36–128) days. All non-smokers at birth utilized NRT and financial incentives, and 9/15 (60%) utilized counseling. Four (9%) were abstinent at 12-week postpartum. Median cigarettes smoked/day reduced from baseline to delivery (10(6–20) to 1(0-6) p =< 0.001). Women who quit smoking had more education (72% vs. 33% p =< 0.02), completed more CO samples (median (IQR) 101(59–157) vs. 2(0–20) p =< 0.001), and received more incentives (median (IQR) $909($225–$1980) vs. $34($3–$64) p =< 0.001). Intervention acceptability was rated favorably by participants (9 items rated 0–10 with scores >5 considered favorable).

**Discussion:**

This study demonstrated the feasibility and acceptability of a consumer-informed, non–face-to-face intensive tobacco treatment, highlighting the potential of remotely delivered technology-based CM to reduce the health impact of tobacco smoking in high-priority populations. The intervention demonstrates scale-up potential. Future studies should extend treatment into the postpartum period, utilizing new technologies to enhance CM delivery and improve counseling provision and partner support.

**Clinical trial registration:**

https://www.anzctr.org.au/Trial/Registration/TrialReview.aspx?id=374196, ACTRN1261800056224.

## Introduction

Alcohol and other drugs (AODs) use, including tobacco use, are some of the most preventable causes of maternal and fetal harms in pregnancy (1, 2). with many women ceasing substance use prior to or early in their pregnancy (3, 4). Pregnant women who continue to smoke tobacco are four times more likely than non-smokers to use other illicit substances concurrently (5), and up to 95% of pregnant women in AOD treatment also smoke tobacco (6, 7). Many reduce or abstain from other substance use but continue to smoke tobacco throughout pregnancy (8–10).

High tobacco smoking rates in maternal substance use populations are influenced and perpetuated by social and physiological factors. Challenging psychosocial circumstances including trauma, stigma, intimate partner violence, and child protection concerns often heighten symptoms of stress or anxiety (11–16), and tobacco smoking is commonly perceived to relieve these (17). Concurrent mental illness is common and is associated with compulsive tobacco use, elevated nicotine dependence, and relapse after quitting (18, 19). Smoking tobacco is known to potentiate the psychoactive effects of some substances or moderate withdrawal from others, increasing the likelihood of use and level of dependence on either (20, 21). Additionally, nicotine metabolism escalates by up to 60% during pregnancy and may cause an increase in prenatal tobacco consumption, particularly in those who are already heavily nicotine dependent (15).

Owing to the complex nature of their circumstances and the challenges associated with substance use treatment, tobacco smoking cessation often receives minimal attention from pregnant women or their healthcare providers (22–25). A documented lack of evidence-based interventions able to reduce tobacco smoking in this group also exists (26, 27).

### Tailoring a tobacco treatment to pregnant women with substance use concerns

To address the high prevalence of tobacco smoking and current treatment deficiencies, multicomponent interventions are required. These should support nicotine withdrawal and accommodate women's competing and complex psychosocial needs, whilst being informed by those with lived and clinical experience (18, 28, 29). The following treatments show potential, particularly when offered in combination, and were presented to clients and clinicians of antenatal facilities that support maternal substance use for feedback (30).

#### Contingency management

Contingency management is a behavioral therapy that provides financial or prize-based incentives in return for biochemically verified abstinence and/or reduction of substance use. The positive reinforcement of behavior change can increase cessation rates across a variety of substances, including tobacco (31). Smoking cessation can be verified by measuring exhaled carbon monoxide (CO) levels in the breath and requires verification at least daily.

A 2017 Cochrane review cited high-quality evidence that contingency management was the most effective treatment for pregnant women when compared to alternative interventions (RR 2.36 95%CI 1.36 to 4.09; 4 RCTs, *N* = 212) (32). There is currently less evidence in maternal substance use populations, with only two studies reported in a 2021 review of tobacco treatments specifically for this population (26). Both trialed contingent incentives in pregnant women with opioid-use disorder. One was unpublished due to null findings. The other reported abstinence in 31% (31/42) of treatment participants at the end of the 12-week treatment, although these rates decreased significantly by 3-month post-treatment (33). Given its promise more broadly, additional evidence for contingency management in pregnant women with substance use concerns is required.

#### Pharmacotherapy

Nicotine replacement therapy (NRT) can improve tobacco outcomes in non-pregnant AOD treatment clients (34). Its efficacy for pregnant women is less certain (35) and may be due to a lack of adherence potentially because recommended doses are too low to counter increased nicotine metabolism during pregnancy (15).

#### Counseling

Evidence suggests that counseling-based interventions can increase tobacco cessation in late pregnancy when compared to usual care (32) although they appear less successful as a stand-alone strategy for pregnant women with co-occurring substance use (36–38). Counseling combined with pharmacotherapy enhances rates of tobacco abstinence for people in AOD treatment (39), and when combined with behavioral therapies such as contingency management improves cessation outcomes in pregnant women (32).

#### Support for partners who smoke

The smoking status of a woman's partner can negatively impact smoking behavior changes during pregnancy (40, 41). Although the effectiveness of encouraging partners to support smoking cessation during pregnancy is inconclusive (42), the provision of evidence-based support by antenatal healthcare providers to address partners' or other household members' smoking has been recommended (43).

#### Consumer and clinical input

Data from qualitative interviews with women who attended antenatal substance use facilities (consumers), and from surveys with their clinicians, suggested that the major barriers to treatment were women stopping multiple substances concurrently, difficulties coping with stress, and the influence of partners who smoke. Clinicians supported contingency management as a treatment approach, and consumers agreed that being rewarded may be helpful, providing the potential for misreporting cessation was minimized. NRT use had negative connotations, particularly around side effects and costs of treatment, although both groups conceded its importance in treatment. Service consumers identified that non-judgmental intervention delivery, education, and motivation were essential components of effective counseling (30).

Based on this study and the aforementioned research findings, the Incentives to Quit Tobacco in Pregnancy (iQuiP) intervention was developed. Its feasibility was assessed when implemented in government-funded public antenatal services that support women who use substances.

## Methods

### Study design and setting

This was a single-arm pre-post feasibility study with full details, methods, and analysis detailed in a published protocol (44). The study was conducted in the antenatal facilities of three major tertiary referral hospitals in NSW and Victoria, Australia. These were integrated AOD and antenatal services that employed nursing staff/midwives, obstetricians, and registrars, as well as drug and alcohol nursing, allied health, and addiction medicine specialists, and provided specialized treatments to improve the outcomes of women and their babies who attended.

The study was registered prospectively with the Australian New Zealand Clinical Trials Registry (Reference: ACTRN12618000576224).

#### Participants and recruitment

Women aged 16 years and over and <33-week gestation who smoked tobacco daily and attended participating antenatal services were invited by antenatal clinicians to enroll anytime from the confirmation of pregnancy to 32-week gestation. Research staff organized face-to-face appointments with women to gain informed consent and provide appropriate education and instruction on contingency management procedures and the use of NRT (See “CO monitoring & NRT guide for participants” in Supplementary material).

#### Intervention

The intervention provided financial incentives for each instance of CO-verified smoking abstinence or reduction, NRT for women and their smoking partners (or other household members who smoked tobacco), and behavioral counseling from study enrolment until delivery, although none were compulsory. Although not considered part of the intervention, good clinical judgement endorsed the need for NRT and counseling to be offered post-treatment from delivery to 12-week postpartum for relapse prevention.

Flexible, non–face-to-face delivery of all study components (excluding informed consent and non-research visits) was arranged to accommodate the competing needs and social backgrounds of participating women as well as a lack of dedicated space at site hospitals. This was achieved using internet-based contingency management methods including electronic retail gift cards as incentives, mail delivery of NRT, and telephone-based counseling and intervention support.

#### Contingency management

Internet-based contingency management is a validated method that allows individuals to self-monitor exhaled CO (45). Women were provided with a portable Bedfont Micro+ Smokerlyzer to assess CO and asked to record their sample-taking using a video-enabled internet device, usually their own smartphone or a study-supplied tablet. A CO cutoff of ≤ 5 parts per million (ppm) was adopted to define abstinence, based on a recommended cutoff of 3 ppm for pregnancy (46) with an allowance for secondary tobacco smoke exposure (47).

Women submitted their CO sample result and timestamped video recording for confirmation by research staff to a Research Electronic Data Capture (REDCap) database (48) via a short survey emailed prior to each expected test. Once submitted, the survey immediately returned an automated and personalized message based on the supplied CO results. For negative samples (those ≤ 5 ppm), a congratulatory message including the incentive earned, accumulated incentive total, and potential future earnings provided immediate reinforcement of behavior change. For positive samples (those >5 ppm), an encouraging message with an accumulated incentive total and potential future earnings was supplied. Samples missed were presumed positive. The process took 2–3 min to complete per sample.

An incentive schedule based on positive reinforcement principles to maximize behavioral change was used (49), with escalating incentives and a CO-sampling regime that reduced in frequency as the duration of abstinence increased (see “Contingency management schedule” and “Incentive payment schedule” in Supplementary material). The schedule specified the number, timing, and earning potential of CO samples in three phases of continuous reinforcement: reduction or shaping, abstinence, and thinning. Incentives for abstinence ranged from $A3.00 to $A20.00 per sample, whereas incentives for smoking reductions were fixed at $A2.50 per sample. Several non-incentivised CO samples were provided prior to contingency management commencing for training and baseline data. The schedule included a contingency reset to encourage abstinence after relapse, whereby positive samples received no incentive, and the value of the next negative sample was reset to the initial rate of $A3.00. After two consecutive negative samples, the incentive reverted to its pre-reset value. Incentives could be accumulated and reimbursed on request.

#### Pharmacotherapy

Women were offered nicotine patches and all oral forms of NRT available in Australia (gum, spray, inhalator, mouth spray, and lozenge) at their baseline visit. They were encouraged to try each to determine their preferred type and use as much as needed to control their urges to smoke (50). Oral NRT was initially recommended for women with low-level nicotine dependence and combination NRT therapy for those who reported heavy or overnight smoking. These were based on the Royal Australian College of General Practice smoking cessation guidelines (51) that endorse the use of short-acting NRT to avoid high levels of nicotine in fetal circulation. Combination NRT was advised if needed to control withdrawal symptoms.

NRT was also provided to women for their partners and/or other household members who smoked. Written and verbal education about NRT use, harm reduction, and safety were provided.

#### Behavioral counseling

A counseling guide was developed based on motivational interviewing and cognitive behavior therapy. The content was women-centered and personalized, focussing on tobacco only. It provided education and strategies to increase motivation, encourage abstinence, and promote relapse prevention (see “Counselor's tobacco treatment guide” and “Participant guide” in Supplementary material). A total of 30 min sessions were delivered by telephone by a diploma-qualified counselor with 20+ years of AOD and tobacco treatment counseling experience. Counseling was offered and encouraged during weekly research calls, although uptake, like other treatment components, was optional.

### Outcomes and data collection methods

The primary outcome was intervention feasibility, determined by the proportions of women enrolled and followed-up at 12-week postpartum and the uptake of individual treatment components. Secondary outcomes assessed participant-reported acceptability and treatment efficacy. This was determined by CO-verified 7-day point prevalence abstinence at birth, which marked the end of treatment, and was determined by the last CO sample provided within 7 days of delivery. Other efficacy outcomes were the length of abstinence and the self-reported reduction in cigarettes smoked from baseline to the last treatment engagement. All outcome variables are described in **Table 2**.

To characterize the sample, demographic (age, cultural status, education, income, relationship status, living arrangements, and gestation) and tobacco smoking characteristics (current vs. previous smoking levels, cigarettes smoked per day, time to first cigarette, CO ppm, previous quit attempts, and household smoking numbers) were captured. Nicotine dependence was determined by the Heaviness of Smoking Index, based on client-reported time to first cigarette and the number of cigarettes smoked per day (52).

Changes in second-hand smoke exposure were assessed by asking how women manage smoking in their homes and vehicles. Response items for homes were: people can smoke anywhere; smoking is allowed in only some rooms inside; smoking is allowed just outside (e.g., outside door, on veranda); or, no smoking is allowed inside and no smoking just outside. Response items for vehicles were: people can smoke in the car whenever they want to; no smoking inside the car when children are present; or, no smoking inside the car ever. Participants were asked at baseline and every 4 weeks during weekly research calls (see Data collection below).

Substance use and quality of life were assessed using the Australian Treatment Outcomes Profile (ATOP) (53), a validated instrument capturing client-reported substance use, health, and wellbeing. Mental health was assessed using the Patient Health Questionnaire (PHQ-9) (54) and the Generalized Anxiety Disorder questionnaire (GAD-7) (55), both validated to assess previous fortnight symptom frequency. The Childhood Trauma Questionnaire (CTQ) (56), a validated non-invasive self-report measure, recorded trauma history.

Intervention acceptability was assessed using a 9-item questionnaire adapted from previous internet-based contingency management studies (45). Respondents rated the effectiveness, convenience, and helpfulness of the intervention, and its components using a 10-point visual analog scale with scores >5 considered favorable. Feedback on the usefulness of counseling sessions was also sought on the same rating scale.

### Data collection

Data were collected from interviews at baseline and 12-week postpartum, and during weekly research calls made for the duration of study engagement by research staff. These calls incorporated the collection of smoking-related data including smoking status, previous 7-day tobacco use, 7-day NRT use, and second-hand smoke exposure. They also encompassed intervention management, addressing additional needs or concerns, resupplying NRT, and reviewing incentives earned and paying them out on request. Counseling sessions were also encouraged and organized as required. These calls typically took approximately 20 min and women received a $A20 voucher for the completion of weekly data collection.

### Statistical analysis

An intention-to-treat analysis was conducted. Descriptive statistics summarized baseline demographic, clinical and smoking characteristics, and weekly survey data. Frequencies (N) and percentages (%), mean (M) and standard deviation (SD), or median (Mdn) and inter-quartile range (IQR) were reported as appropriate unless otherwise specified. Differences between women smoking and not smoking at birth were calculated using independent sample *t*-tests or Mann–Whitney U-tests for numerical variables, and Fisher's exact test for categorical variables. Sign tests determined changes in the management of smoking in homes and vehicles. Analyses were programmed using SPSS v28, SAS 9.4, and R 4.2.0.

## Results

### Recruitment and participant characteristics

**Figure 1:**
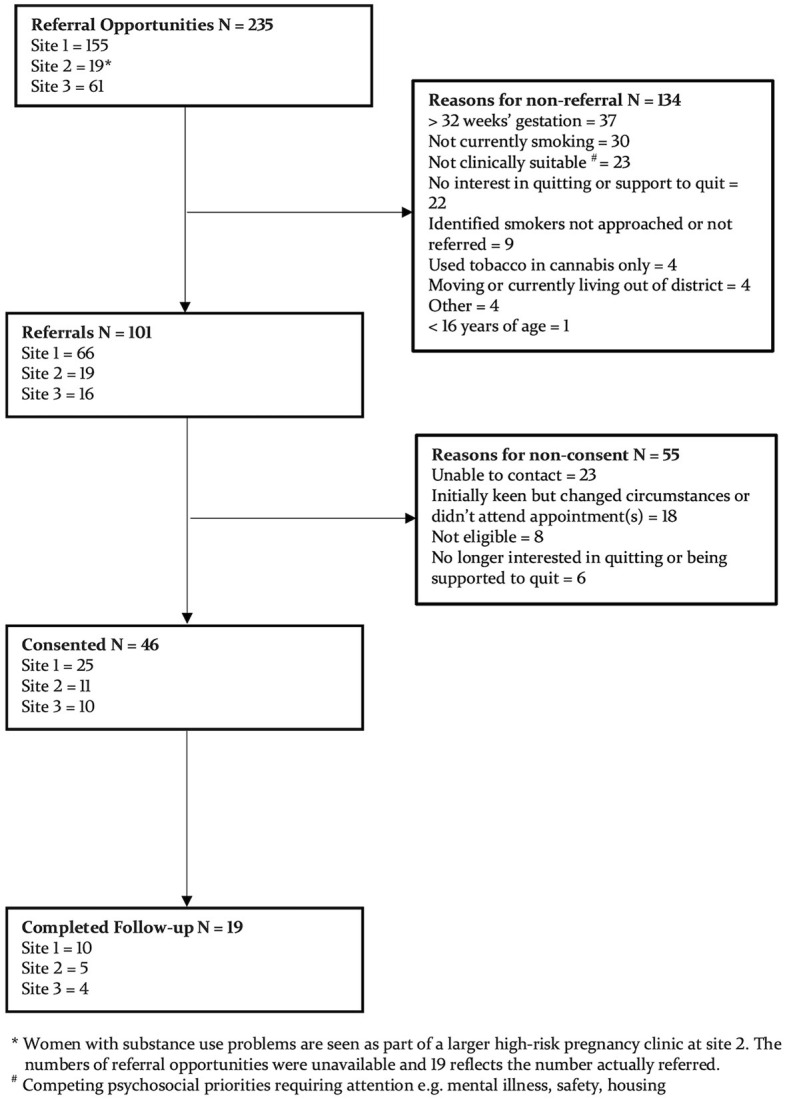
Participant flow through the study.

Screening of 101 women and consent of 46 occurred from July 2018 to June 2020 (see Figure 1), with recruitment commencement and length of recruitment varying between sites. Median (IQR) treatment engagement is 75 (42–122) days, determined by the number of days between consent and the last data collection call prior to delivery. Seventy-eight percent (36/46) took at least one weekly research call, with a median (range) of 9 (1-22).

Baseline characteristics are described in Table 1. Additional results at baseline indicated that 55% (24/46) had reduced cigarette consumption whilst pregnant, and 37% (17/46) were currently trying to stop. Of the 43 women who responded to previous quit attempt questions, 14% (6/43) had never tried quitting tobacco. Of those who had tried quitting tobacco, 54% (20/37) remained abstinent for 3 months or more, and 38% (14/37) of these attempts were during pregnancy. Most had used NRT to assist quitting (65%; 24/37) followed by vaporized nicotine products (12/37; 32%), Quitline (22%; 8/37), and prescription medications (varenicline and bupropion) (14%: 5/37).

**Table 1:** Demographic, smoking, and substance use characteristics at baseline (*N* = 46).

**Demographic and tobacco smoking characteristics**	**Total group (*N =* 46)**	**Not smoking at birth (*N =* 15)**	**Smoking at birth (*N =* 31)**	***P*-value**
Age, years M (SD)	31.3 (6.2)	32.6 (5.5)	30.6 (6.5)	0.30
Aboriginal or Torres Strait Islander, yes *N* (%)	10 (22%)	3 (20%)	7 (23%)	1.00
Education, high school only *N* (%)	26 (59%)	5 (33%)	21 (72%)	0.02^*^
Income, government support *N* (%)	33 (75%)	13 (87%)	20 (69%)	0.28
Relationship status, single *N* (%)	20 (45%)	7 (47%)	13 (45%)	1.00
Living arrangements, renting *N* (%)	28 (64%)	9 (60%)	19 (66%)	0.75
Gestation at screening M (SD)	22.3 (5.8)	20.7 (5.3)	23.2 (6.0)	0.16
Current smoking, same or more than pre-pregnancy levels *N* (%)	20 (46%)	7 (47%)	13 (45%)	0.76
Cigarettes smoked/day Mdn (IQR)	10 (6–20)	7 (3–20)	10 (7–20)	0.22
Time to the first cigarette, within 30 min of waking *N* (%)	25 (58%)	6 (40%)	19 (68%)	0.11
**Heaviness of Smoking Index classification**				
Low *N* (%)	29 (63%)	10 (66%)	19 (61%)	0.77
Moderate *N* (%)	13 (28%)	5 (33%)	8 (26%)	0.85
High *N* (%)	0	0	0	
CO ppm at baseline Mdn (IQR)	15 (8–26)	13 (9–29)	15 (8–25)	0.84
Previous quit attempts, yes *N* (%)	36 (86%)	13 (93%)	23 (82%)	0.65
Smokers in household, > 1 *N* (%)	33 (75%)	11 (73%)	22 (76%)	1.00
Smoking allowed inside a house, yes *N* (%)	12 (28%)	3 (20%)	9 (31%)	0.50
Smoking allowed inside the car including when children present, yes *N* (%)	32 (74%)	10 (71%)	22 (76%)	1.00
**Health and wellbeing**				
Anxiety, number in moderate to severe category, *N* (%)^a^	13 (28%)	6 (40%)	7 (30%)	0.73
Depression, number in moderate to severe category, *N* (%)^b^	14 (38%)	8 (57%)	6 (26%)	0.09
Experienced childhood trauma, *N* (%)^c^	17 (45%)	7 (47%)	10 (44%)	1.00
Self-reported quality of life, M (SD)^d^	6.9 (1.8)	6.1 (1.4)	7.3 (1.9)	0.04^*^
Self-reported mental health M (SD)	5.6 (2.2)	5.6 (1.9)	5.6 (2.5)	0.91
Self-reported physical health M (SD)	5.9 (2.3)	5.1 (2.0)	6.4 (2.4)	0.07
**Self-reported substance/s of concern**				
Cannabis *N* (%)	28 (61%)	9 (60%)	19 (61%)	0.93
Amphetamines *N* (%)	11 (24%)	5 (33%)	6 (19%)	0.46
Alcohol *N* (%)	9 (20%)	2 (13%)	7 (23%)	0.70
Benzodiazepines *N* (%)	3 (7%)	2 (13%)	1 (3%)	0.24
Opioids *N* (%)	3 (7%)	1 (7%)	2 (6%)	1.00
**Therapeutic drugs**				
Opiate agonists *N* (%)	7 (15%)	4 (27%)	3 (10%)	0.19

a Identified using GAD-7, a brief screening measure for symptoms of anxiety.

b Identified using PHQ-9, a brief screening measure for symptoms of depression.

c Self-reported moderate to severe experience of emotional, physical or sexual abuse and/or emotional or physical neglect as per Childhood Trauma Questionnaire (CTQ). *N* = 38.

d Self-rated score on 0 to 10 visual analog scale, where 0 = poor and 10 = good.

* Indicates results significant at the 0.05 level.

### Feasibility and acceptability

Table 2 presents outcome results indicating a recruitment rate of 46% (46/101) and a retention rate of 41% (19/46) at 12-week postpartum follow-up. Most women (70%; 32/46) utilized CM, completing a median of 16 (0–72) CO samples and earning an average of $430. Details and breakdown of incentives earned and CO-sampling adherence are available in Supplementary material.

**Table 2:** Primary and secondary outcome variables and results (*N* = 46; all assessed at birth unless otherwise stated).

**Primary outcome**		
**Feasibility**		
Recruitment rate (number recruited/number screened, %)	46/101	46%
Intervention retention rates (number completing follow-up at 12-week postpartum/number recruited, %)	19/46	41%
CO sample rate (actual COs completed/total possible CO's, %)	2,030/3,545	57%
Number of counseling sessions completed (Mdn, range)	1	0–10
Women using NRT (reported >1 week use, %)	32/46	70%
Adherence to NRT (number who requested NRT after initial supply, %)	29/46	63%
Partners/household members receiving NRT (number, %)	10/46	22%
**Secondary outcomes**		
**Changes in tobacco smoking**		
Number of verified abstinent days ( ≤ 5 ppm; actual number of days/total possible number of days, %)	1,566/3,545	44%
Self-reported 7-day point prevalence verified by CO at birth ≤ 5 ppm (N%)	15	33%
Self-reported reduction in the number of cigarettes smoked/day in past 7-day baseline to last contact (M, p-value)	13 vs. 3	< 0.001
Changes in the management of smoke-free homes (number positive vs. negative vs. no change, *p*-value)	14 vs. 1 vs. 13	< 0.01
Changes in the management of smoke-free vehicles (number positive vs. negative vs. no change, *p*-value)	18 vs. 1 vs. 9	< 0.01
**Treatment acceptability (*****N*** = **23)**^a^	***Mdn***	***IQR***
Ease of participation	7	(6–10)
Intervention helpfulness	8	(7–10)
Convenience of taking part	8	(7–9)
Opinion of using CO meter	6	(5–9)
Opinion of earning financial incentives	10	(5–10)
Effectiveness of incentives	8	(5–10)
Effectiveness of telephone-based support	7	(4–10)
Effectiveness of NRT	9	(7–10)
Fairness of a government-funded service using incentives to aid smoking cessation	8	(7–10)

^a^Rated using a 10-point visual analog scale with scores >5 considered favorable.

Nearly all women (98%; 44/45) accepted the NRT offered at baseline. Just over half (52%; 24/46) used patches, 46% (21/46) used mouth spray and gum, 41% (19/46) used inhalators, and 24% (11/46) used lozenges. Half (50%; 23/46) of the women attended at least one counseling session, and of these, 43% (10/23) attended two or more. The median usefulness of counseling was 9 (7–10) out of 10. Twenty-three women completed the treatment acceptability questionnaire, with median endorsements for all items >5, indicating the overall acceptability of the intervention and its components.

### Treatment effectiveness

Treatment effectiveness outcomes are outlined in Table 2. Seventeen women (37%) self-reported 7-day point prevalence abstinence at birth. Fifteen (33%) provided CO verification of abstinence with a verified median period of abstinence of 65 (36–128) days. Median cigarettes smoked per day reduced from baseline to birth or last contact by 90% [10 (6–20) to 1 (0–6) *p* = < 0.001]. Four women (9%) reported not smoking at the 12-week postpartum follow-up.

Baseline characteristics of non-smokers and smokers at birth are compared in Table 1. Of the non-smokers at birth, 100% (15/15) utilized NRT and financial incentives, and 60% (9/15) utilized counseling. As expected, non-smokers completed more CO samples (Mdn (IQR) 101 (59–157) vs. 2 (0–20) *p* = < 0.001) and received more financial incentives (Mdn (IQR) $A909 ($A225–$A1980) vs. $A34 ($A3–$A64) *p* = < 0.001) than those that continued to smoke at birth. They were also more likely to utilize NRT (100% vs. 55%, *p* = 0.002), though median counseling attendance and NRT uptake by partners were not significantly different (1 (0–2) vs. 0 (0–1) *p* = 0.36 and 33% vs. 16%, *p* = 0.26, respectively).

## Discussion

This study evaluated the feasibility and acceptability of a non–face-to-face delivery of tobacco treatment incorporating a novel combination of contingency management, NRT, counseling, and partner support for pregnant women who smoked tobacco and were having treatment for other substance use problems. For a group that is typically challenging to enroll and retain in research, and for whom smoking cessation is often not prioritized, half the potentially interested clients were enrolled, and almost half of those who did enroll were retained until the 12-week postpartum follow-up. Comparisons with studies in similar populations are difficult because treatment types, intensities, and durations differ. For example, Fallin-Bennett et al. (57) retained 66% (34/50) of pregnant OAT clients enrolled in a 12-week education and support-based treatment with no follow-up, whilst Ainscough et al. (58) retained 25% (10/40) after a 6-week contingency management intervention for non-pregnant OAT clients. Recruitment and retention in the current study may have been hindered by the perceived burden of self-recording and uploading CO samples using multiple platforms. Newly developed application-based software allows smartphones to connect with personal Bluetooth-enabled CO monitors and assess samples whilst verifying user identity with facial recognition. This technology could facilitate a less onerous process and potentially improve retention rates (59, 60).

The uptake of treatment components, apart from partner/household support, was acceptable, and small adjustments could improve future delivery and utilization rates. The contingency management component was utilized by 70% of women with CO sample completion rates for those aiming for abstinence comparable to rates in general populations (81% vs. 82%) (49). The uptake of NRT was greater than expected, given the doubts raised by women and clinicians who informed the intervention's development. Women were encouraged to trial all NRT forms and use combination therapy where necessary. The provision of harm reduction education, instructions for correct use, and follow-up support were also important for enhancing initiation and adherence to NRT (50).

Half the women attended one or more counseling sessions, with uptake potentially hampered by the financially reimbursed research support calls made by staff with tobacco treatment training. Having two overlapping roles was confounding but necessitated by the approving ethics committee, which required counseling to be separated from research-related tasks. Anecdotally, several women refused counseling citing the support provided by research calls as being adequate. Helpfulness ratings suggest that counseling was valuable, and its ability to enhance the effectiveness of other behavioral and/or pharmacological measures is well-known (61).

Uptake of NRT by partners/household members was low (22%) given that 75% of women reported living with other smokers. The NRT provision for this subgroup lacked the same instruction and support that enrolled participants received, and data collection on utilization was poor. Future studies might consider delivering these directly to partners/household members to improve uptake and reporting (42), whilst qualitative interviews with women and partners/household members could determine whether such support is important for future studies. Overall, the satisfactory uptake of treatment components other than partner NRT, the high level of treatment engagement by women, and the favorable acceptance ratings all indicate the potential for intervention implementation more broadly in healthcare settings.

Preliminary intervention effectiveness was evidenced by 33% (15/46) of women being verified as smoke-free at birth following a median 9-week period of abstinence, an achievement that could improve maternal and fetal outcomes (62, 63). Comparisons with earlier studies are difficult as outcomes may be mediated by treatment duration, intensity of support provided, or differences in substances used. For example, in a study targeting pregnant women on OAT, Fallin-Bennet et al. (57) reported 4% (2/50) abstinence after a 12-week treatment, whilst Tuten et al. (33) reported 31% (13/42) abstinence at week 12 of a randomized contingency management intervention, but no abstinence at 6-week postpartum.

Abstinence in the current study fell to 9% at 12-week postpartum, concurring with evidence that nearly half of those participating in smoking cessation trials during pregnancy will return to smoking by 6-month postpartum (64). The postpartum decline in abstinence was almost certainly influenced by the withdrawal of support and financial incentives (65) although recent findings suggest that abstinence generally extends further than incentives in pregnant and non-pregnant populations (66). Other factors thought to impact postpartum abstinence were the strict criteria utilized for determining abstinence as well as restrictions on conducting CO tests during the COVID-19 period and poor staff retention, both of which interfered with the follow-up of participants.

Further significant behavior changes during treatment were reductions in cigarette consumption, and positive changes in the management of tobacco smoking in households and vehicles, creating a much-needed reduction in secondhand smoke exposures. Women who did not smoke at birth were more likely to have a high-school education but scored lower on quality-of-life measures. All scores come from a sample underpowered to detect such differences, and this may explain these anomalies. Moreover, the psychometrics of the ATOP-derived quality-of-life data suggest that the between-group differences were not clinically significant.

### Strengths and limitations

The intervention incorporated several novel features that may have contributed to its feasibility and acceptability. The non–face-to-face delivery of intervention components reduced the time and financial burden on women to attend extra and/or longer clinic appointments. The autonomy to choose individual and complementary evidence-based treatments is not a common research practice, but one that can improve treatment adherence in AOD treatment populations (67). Finally, the intensity of support provided to women was exceptional. Tobacco treatments for general populations are often brief and/or contain one or two treatment elements with minimal clinical contact (68). Interventions with two or more components or that combine intensive behavioral support and pharmacotherapy can increase smoking cessation outcomes compared to single-component interventions (28, 69). Additionally, multi-faceted treatments, regular follow-up, and opportunities for supplemental help have been deemed necessary components of tobacco treatment by pregnant women with substance dependence (57, 70). Identifying an optimally effective, acceptable, and affordable level of support, however, requires further investigation.

The study findings should be viewed in the light of some methodological limitations including the interpretation of treatment effectiveness outcomes. Although encouraging, they are exploratory, and caution should be considered in the context of the single-arm design and small sample size. The use of a single arm study design was considered appropriate given the evidence-base for individual treatment components and nature and size of the study population.

Ceasing tobacco treatment at birth was a major limitation. Budgetary constraints were a key consideration in this decision; however, investigators felt it clinically appropriate to offer NRT and counseling during the follow-up period. As weekly calls to prompt women were no longer made, the uptake of these resources was poor. Future studies should extend treatment, including support for partners who smoke, into the challenging postpartum period to create long-term change rather than a temporary suspension of smoking during pregnancy (71). Improvements to programme delivery and the associated cost efficiencies could potentially make such extensions economically viable.

Another consideration was the monetary reimbursement of research calls. Whilst this may have hampered the uptake of counseling, it may also have contributed to the intervention's success as incentivised participation in substance use treatment increases attendance and also post-treatment abstinence outcomes (72). Future studies should consider combining the counseling and intervention support roles into one incentivised weekly call to increase participation and the quantity of counseling delivered. The cost of this should be balanced with incentives for abstinence to optimize motivation and programme affordability.

A final limitation was the exclusion of women over 32-week gestation. An eligibility cutoff of 36-week gestation was vetoed by the approving ethics committee due to their concerns that tobacco treatment would lack benefit after this time. These claims were unsubstantiated and significantly impacted recruitment by excluding women who typically attend antenatal treatment later in pregnancy (73).

### Future directions

The current study has demonstrated potential for scaling up to a fully powered clinical trial. Several recommendations have been identified to increase its impact and improve treatment outcomes.

Whilst contingency management shows promise for increasing tobacco abstinence rates (66) difficulties in funding and implementing programmes in real-world settings threaten its translatability. Capitalizing on dedicated mobile software and automated systems will improve the user experience and eliminate manual handling, although doing so may increase project lead times and set-up costs. The feasibility of non–face-to-face delivery allows for centralized implementation with a single person or small team able to oversee intervention management and cater to a broader geographical area.

The level of incentives used in the current study may be prohibitive if the intervention was scaled up. Whilst current average earnings of $A430 are comparable to other smoking-based contingency management programmes in high-income countries (74), higher financial amounts do not always equate to higher rates of abstinence (66, 75). Small financial incentives have improved abstinence amongst socioeconomically disadvantaged individuals who smoke tobacco (76), although an optimal amount that cost-effectively promotes change has not been identified (75). An analysis of treatment costs vs. healthcare savings in the Australian context (77) is required to assist decision-making around the economic viability of future studies.

The inclusion of economic evaluation data was originally planned for the current study, along with qualitative outcomes of patient- and clinician-considered treatment acceptability and a comparison of tobacco-related maternal and neonatal characteristics to assess whether smoking reductions led to improvements in these areas. The scope of these outcomes has precluded their inclusion here, and results will be reported in separate publication(s).

## Conclusion

A multicomponent intervention incorporating contingency management, NRT, counseling, and partner/household support to address tobacco dependence in pregnant women with substance use concerns appears to be feasible and acceptable. The intervention used non–face-to-face delivery and offered women autonomy over their treatment. The results suggest an opportunity for future improvement and scale-up with extension into the postpartum period essential. The intervention will benefit from new technology to improve contingency management processes and an economic evaluation to inform the economic feasibility of larger-scale programmes. Treatments that address tobacco smoking in nicotine-dependent pregnant women who use other substances are important for reducing the ongoing cycle of tobacco use that impacts the health and wellbeing of the community and future generations.

## Data availability statement

The raw data supporting the conclusions of this article will be made available by the authors, without undue reservation.

## Ethics statement

The studies involving human participants were reviewed and approved by Hunter New England Human Research Ethics Committee (Reference: 17/04/12/4.05) Aboriginal Health and Medical Research Council of NSW (Reference: 1249/17). The patients/participants provided their written informed consent to participate in this study.

## Author contributions

Study conceptualization, funding, and design were led by AD and GG with input from MJ, ABr, ABa, BB, PH, JA, and NP. The study was conducted by MJ and JB, with resources provided by AD and YB. Formal analysis was conducted by MJ and DB. MJ wrote the first draft of the manuscript. All authors contributed to its revision and approved the submitted version.

## Supplementary material

Supplementary Data Sheet 1 CO monitoring & NRT guide for participants.

Supplementary Data Sheet 2 CO monitoring incentive schedule.

Supplementary Data Sheet 3 Incentive payment schedule.

Supplementary Data Sheet 4 Tobacco treatment manual.

Supplementary Data Sheet 5 Smoking cessation - A guide for participants.
