# Supplementary material for: Tobacco treatment incorporating contingency management, nicotine replacement therapy, and behavioral counseling for pregnant women who use substances: a feasibility trial
Source: Front Psychiatry. 2023 Aug 16;14:1207955. doi: 10.3389/fpsyt.2023.1207955 (PMC10467262; doi:10.3389/fpsyt.2023.1207955)
Supplement: Supplementary Data Sheet 1 — CO monitoring & NRT guide for participants. [file Data_Sheet_1.PDF]

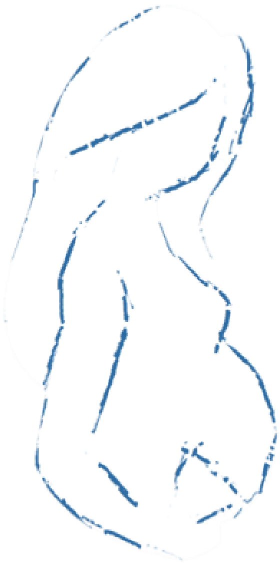

Incentives to Quit tobacco in Pregnancy (iQuiP)

CO Monitoring & NRT

A guide for participants

CO Monitoring

You will be expected to provide Carbon Monoxide (CO) samples regularly while you participate in this study. They are used to verify that you are meeting the CO targets set by the research staff or that you are abstinent from smoking tobacco. Every CO sample submitted that meets the required target and is verified will earn a financial incentive.

CO Monitoring Guidance

In order to provide a CO sample, you will need to record yourself giving a sample using the CO monitor provided by the study.

The following are guidelines to give a CO sample:

1. Turn on CO monitor by pressing the power button once. The display screen will be in **ready mode**
2. Press the female breath test symbol on the screen
3. To cancel the breath test, press
4. When ready, **breathe in deeply** and hold for 15-seconds. The monitor will count down for you and a beep will sound during the last 3 seconds of the countdown.
5. Exhale **slowly** into the mouthpiece, until your lungs are completely empty.
6. The onscreen CO ppm levels will rise and then stop
7. The test is finished when appear on the screen
8. If a high reading has been recorded, you can mute the sound by pressing
9. To repeat breath test, press to return to the home screen and begin from step 3.

Recording the CO sample

Use your mobile phone or other internet enabled recording device (e.g. iPad or tablet) to record your CO sample. The following guidelines must be followed:

1. Video must be recorded using the **Timestamp Camera app** that was downloaded when you joined the study. This marks each video with the time and date of the sample.
2. Use one hand to hold the phone and record the sample, or ask someone to record you, making sure that the following is visible in the video recording:
    a. The display screen in **ready mode** before the sample is given
    b. You **inhaling and holding** your breath for the 15 second countdown
    c. You **blowing** into the monitor
    d. The **results** on the monitor screen

Each video will be watched by a research team member before being verified. If any of these are not in the video, or if there is any doubts about the sample or who is providing it, then you will be

contacted and asked to provide another sample. This sample will be done in front of a research team member using videoconference.

Sending the CO sample to the research team

Before every CO sample that you are required to complete, an email will be sent providing a link to a short survey. The survey contains questions to collect the required information and a link to upload the video recording.

1. **Sample date** – press 'Today' to automatically fill with the current date
2. **Sample time** – if the sample was just completed press 'Now' to automatically fill the current time. If not, fill the time the sample was done.
3. **Have you smoked tobacco today?** – Yes or no
4. **CO sample reading –** fill with the final CO ppm displayed on the monitor
5. **Upload video** – Pick 'choose from file', select the correct video from your photo library and press 'Upload'. This may take a while depending on the size of the video.
6. **Submit** – this will send the information and video to a secure database for the research team to review and verify.
7. **Feedback** – If a negative sample (5 ppm or less) was submitted, a congratulatory message will appear with details about the incentive earned, total incentives to date and what you can expect to earn from your next CO sample. If the sample was positive (over 5ppm) then the message will be an offer of encouragement.

Please note: Each sample has to be verified, so the incentive value will change if it is found to be incorrect or falsified in any way.

1. Read email and press the 'CO Baseline Submission Survey' link

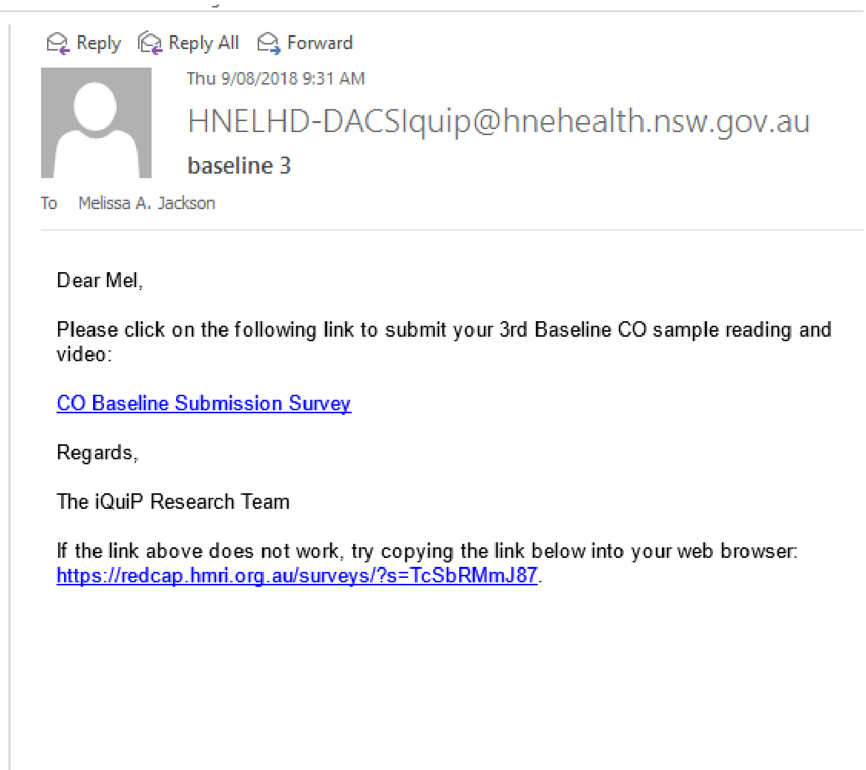

Reply Reply All Forward

Thu 9/08/2018 9:31 AM

HNELHD-DACSIquip@hnehealth.nsw.gov.au

baseline 3

To Melissa A. Jackson

Dear Mel,

Please click on the following link to submit your 3rd Baseline CO sample reading and video:

CO Baseline Submission Survey

Regards,

The iQuiP Research Team

If the link above does not work, try copying the link below into your web browser:
https://redcap.hmri.org.au/surveys/?s=TcSbRMmJ87

2. Complete as follows:
    a. Date - press ‘today’
    b. Time – press ‘now’
    c. Press ‘yes’ or ‘no’
    d. CO Sample reading – this is the CO reading number shown on the monitor e.g. 5
    e. Video upload – press green link to upload video from file

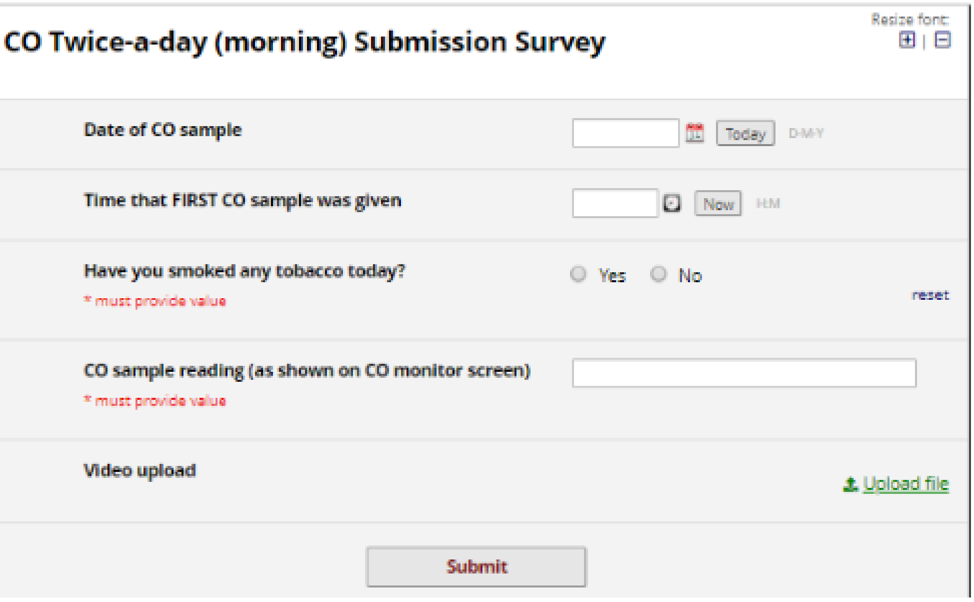

3. Press Browse to be taken to your camera’s photo gallery. Select the correct video and press ‘Upload Document’

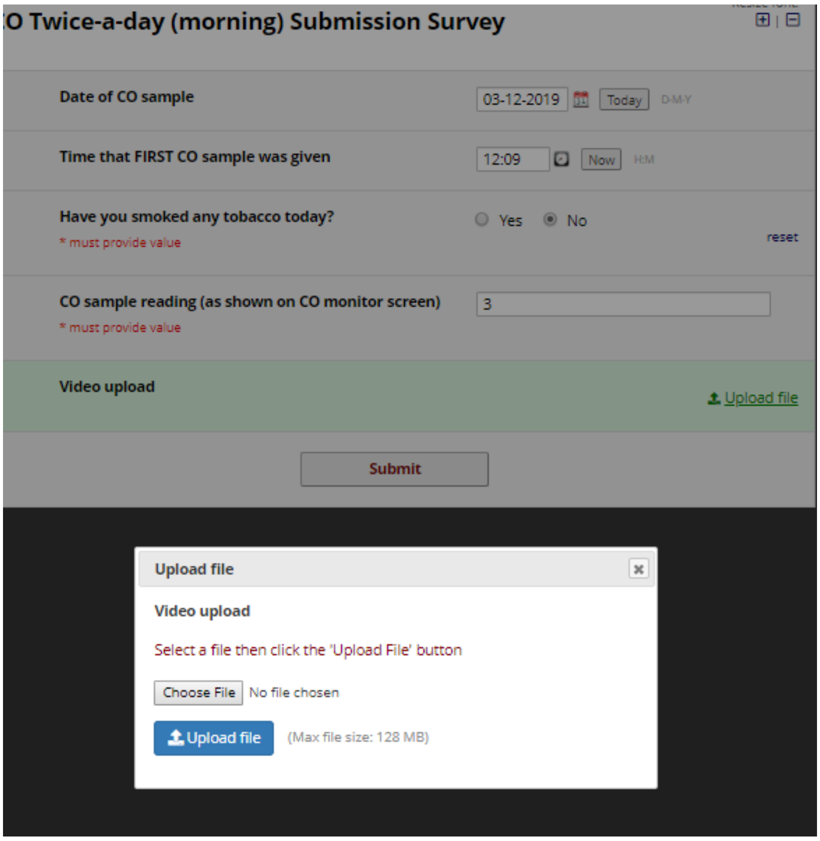

4. The upload process may take a minute or two, depending on the size of the video.

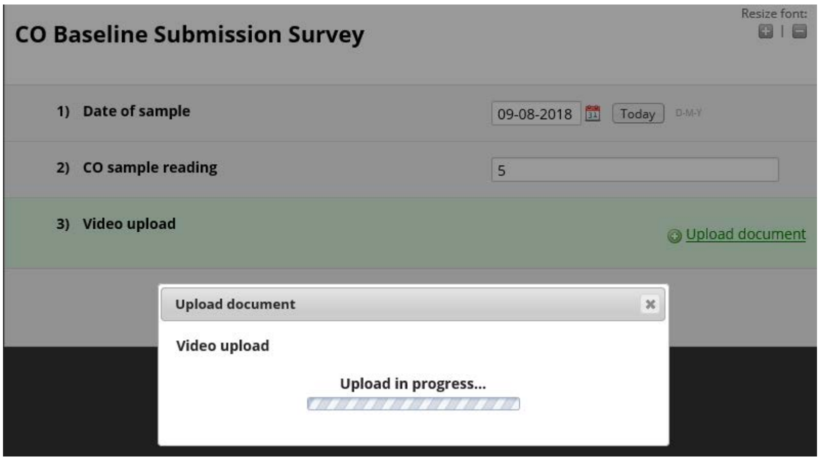

5. When the upload is finished, press 'Submit'

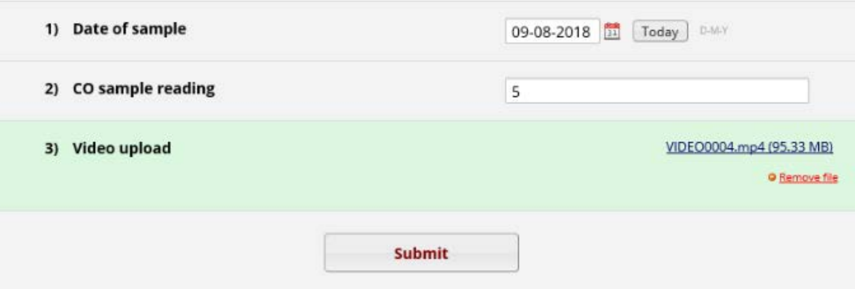

6. Except for the 5 x baseline CO samples, all other samples will provide a message giving feedback about the incentive you have just earned, the total incentives earned and what you will earn for your next negative CO sample

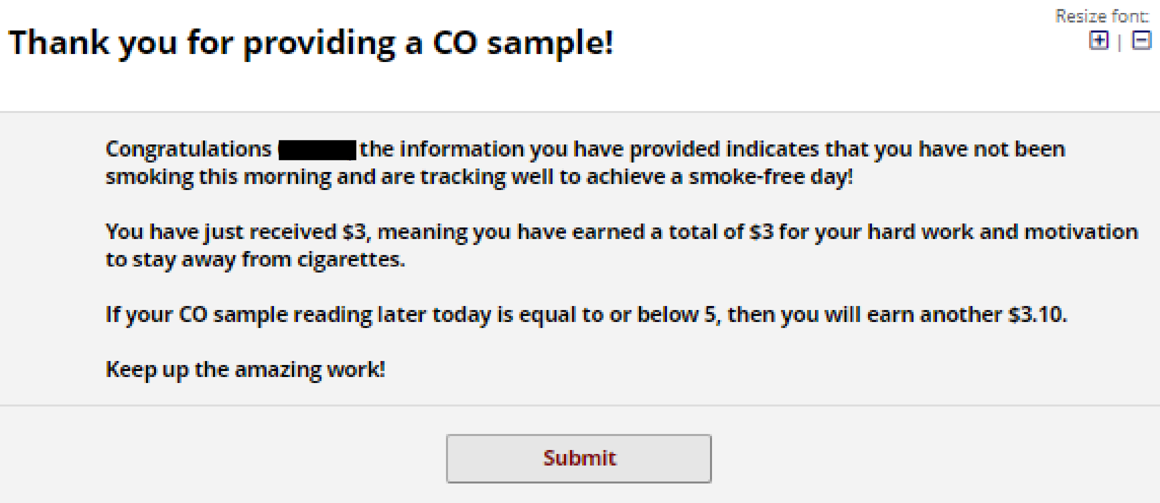

7. Pressing 'Submit' again will shut down the survey.

Close survey

Thank you for submitting your baseline CO reading. Please let us know if you had problems with the testing today.

Enjoy the rest of your day!

The iQuiP research team

Phases of CO Monitoring

There are 5 phases of CO monitoring, each with different CO requirements and incentive values. You may only use one or two of these phases, this will be determined by your motivation and length of time on the program and will be guided by the iQuiP team.

1. Baseline

- Five days of CO readings that provide us with your baseline CO reading.
- No incentives are earned for these samples
- Good opportunity to practice your sampling and recording technique

2. Reduction

- You may decide to reduce your smoking for up to 4 weeks before stopping altogether
- Reduction CO target levels are set by the research team
- Incentives are lower than those earned if you stop
- You can submit up to two samples per day and receive $2.50 for every verified sample that is below the current CO target

3. Abstinence - Twice per day

- Once you have given up, you will be required to submit 2 samples per day for 4 weeks.
- The 2 samples must be at least 8 hours apart
- Each CO sample must be below 6 ppm (5 or under)
- Incentives will start at $3 per sample and increase by $0.10 per sample until a maximum of $20.00 is reached (a full incentive schedule is included at the back of this guide).

4. Abstinence – Once per day

- After 4 weeks of twice daily monitoring, you will reduce to once per day.
- These will need to be completed at different times on different days so that they are not all morning or afternoon or evening
- Each CO sample must be below 6 ppm (5 or under)
- Incentive values will continue from the previous phase

5. Thinning – Every other day

- For the 4 weeks prior to your expected delivery date, monitoring will reduce to every second day
- Incentives will only be paid every second day
- This is to reduce the burden of monitoring and to reduce your reliance on the rewards

Incentive reset

Whenever a CO sample is missed, it is assumed to be a positive, and no incentive will be earned. The incentive for the next negative sample will then be reduced to the starting amount of $3.00. However, to keep motivation high, after two negative samples, the incentive will return to the amount you were on before the missed or positive sample.

Care of your CO monitor

The CO monitor provided to you for the period of the study is on loan only. After the CO monitoring period is over, you will be expected to return it in good, working condition.

While it is in your care, please take notice of the following rules around its use and care:

- The straw-like mouthpieces should be replaced after each use
- Only wipe the instrument and D-piece (that holds the straw mouthpiece) external surfaces with the cleaning wipes provided. The D-piece cannot be sterilised.
- **<u>NEVER</u>** use alcohol or baby wipes containing alcohol or other cleaning solvents containing alcohol as these can damage the CO sensor inside.
- Under no circumstances should the instrument be immersed in liquid or splashed with liquid.
- Replace batteries when indicated by the empty symbol 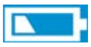
- Replace breath sampling D-piece every 30 days or if visibly soiled or contaminated. The device will give this reminder when the D-piece should be replaced. 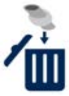

Nicotine Replacement Therapy

Directions for NRT Use

Nicotine patch

- Apply a patch to a clean, dry, hairless part of the skin, preferably when waking up.
- There may be some irritation or itchiness for the first hour or so, but this should resolve itself.
- Move patch position daily if necessary.
- The 16 hour patch releases a constant supply of nicotine for 16 hours. If using 24 hour patches, advise participants to remove before going to bed.
- Overnight patching or double patching may be required for cases of heavy dependence and overnight smoking.

Nicotine gum

- Use in place of having a cigarette, or when a craving hits.
- A variety of flavours exist.
- Chew, Park, Chew:
    - Chew a single piece of gum slowly for approx. 1 minute until the taste becomes strong.
    - Stop chewing, rest or 'park' the gum against your cheek or under your tongue.
    - When the taste fades, chew a few more times until the taste gets strong, then park the gum again.

Lozenges

- Use in place of having a cigarette, or when a craving hits.
- Place a single lozenge in the mouth and allow it to dissolve rather than chew it. This should take approx. 30 minutes.
- From time to time, move the lozenge from one side of the mouth to the other.

Mouth spray

- Use in place of having a cigarette, or when a craving hits.
- Use 1 or 2 sprays each time. Aim to use no more than 4 sprays per hour or 64 sprays per day.
- There are approx. 150 sprays per canister.
- Spray the mist under the tongue or inside the cheek. Don't spray it into the back of the mouth or on the gums as this may give a burning sensation.
- The mouth spray is the fastest acting nicotine replacement therapy product, but the taste can be too strong for some people.

Inhalator

- Take short puffs from the inhalator rather than inhaling (like on a cigarette) as it is important for the nicotine to be absorbed in the mouth.
- Inhaling down the throat may cause throat irritation or feelings of nausea.
- The Inhalator contains 15mg of nicotine. Each cartridge lasts for about 40 minutes with continual use.
