# Supplementary material for: Tobacco treatment incorporating contingency management, nicotine replacement therapy, and behavioral counseling for pregnant women who use substances: a feasibility trial
Source: Front Psychiatry. 2023 Aug 16;14:1207955. doi: 10.3389/fpsyt.2023.1207955 (PMC10467262; doi:10.3389/fpsyt.2023.1207955)
Supplement: Supplementary Data Sheet 2 — CO monitoring incentive schedule. [file Data_Sheet_2.PDF]

Contingency management incentive schedule outlining rationale, duration and procedures for smoking verification

Phase & Rationale	Duration	Procedure
**Baseline**		
– Training and baseline data	– Up to 5 days	– Samples supplied once daily<br>– Not incentivised
**Reduction or shaping**		
– Incentivised reductions in smoking<br>– Encouraged learning and reinforcement prior to abstinence<br>– Optional	– Up to 4 weeks	– Weekly reduction CO targets calculated using baseline CO<br>– Samples supplied up to twice daily (am & pm)<br>– Reduction incentives fixed at $2.50
**Abstinence**		
– Escalating incentive for each verified CO sample ≤5ppm (i.e. negative CO sample)	– Post baseline or reduction to commencement of thinning	– Samples supplied twice daily (at least 8 hours apart) for 4 weeks<br>– After 4 weeks of abstinence, they reduced to once daily<br>– Incentives started at $3.00 & increased by $0.10 for each verified negative sample<br>– Incentives capped at $20.00
**Thinning**		
– Reduced monitoring requirements<br>– Reduced reliance on incentives	– 4-weeks prior to expected delivery date	– Samples supplied every second day<br>– Only applied to those who were abstinent for > 6 weeks
**Contingency Reset**		
– Incentives not provided for missed or positive samples (i.e. those >5ppm)<br>– Value of subsequent negative samples (≤5ppm) will be reduced<br>– Incentive values can be reset to encourage abstinence after relapse	– As needed	– Following a positive sample, the value of the next negative sample was reset to the initial rate ($3.00)<br>– Two consecutive negative samples reverted the next incentive to its pre-reset value
