# Supplementary material for: Tobacco treatment incorporating contingency management, nicotine replacement therapy, and behavioral counseling for pregnant women who use substances: a feasibility trial
Source: Front Psychiatry. 2023 Aug 16;14:1207955. doi: 10.3389/fpsyt.2023.1207955 (PMC10467262; doi:10.3389/fpsyt.2023.1207955)
Supplement: Supplementary Data Sheet 3 — Incentive payment schedule. [file Data_Sheet_3.PDF]

iQuiP Incentive Payment Schedule

Week number	Day 1		Day 2		Day 3		Day 4		Day 5		Day 6		Day 7		Weekly Total	Accumulated Total
**1**	3.00	3.10	3.20	3.30	3.40	3.50	3.60	3.70	3.80	3.90	4.00	4.10	4.20	4.30	51.10	**51.10**
**2**	4.40	4.50	4.60	4.70	4.80	4.90	5.00	5.10	5.20	5.30	5.40	5.50	5.60	5.70	70.70	**121.80**
**3**	5.80	5.90	6.00	6.10	6.20	6.30	6.40	6.50	6.60	6.70	6.80	6.90	7.00	7.10	90.30	**212.10**
**4**	7.20	7.30	7.40	7.50	7.60	7.70	7.80	7.90	8.00	8.10	8.20	8.30	8.40	8.50	109.90	**322.00**
**5**	15.00		15.10		15.20		15.30		15.40		15.50		15.60		107.10	**429.10**
**6**	15.70		15.80		15.90		16.00		16.10		16.20		16.30		112.00	**541.10**
**7**	16.40		16.50		16.60		16.70		16.80		16.90		17.00		116.90	**658.00**
**8**	17.10		17.20		17.30		17.40		17.50		17.60		17.70		121.80	**779.80**
**9**	17.80		17.90		18.00		18.10		18.20		18.30		18.40		126.70	**906.50**
**10**	18.50		18.60		18.70		18.80		18.90		19.00		19.10		131.60	**1038.10**
**11**	19.20		19.30		19.40		19.50		19.60		19.70		19.80		136.50	**1174.60**
**12**	19.90		20.00		20.00		20.00		20.00		20.00		20.00		139.90	**1314.50**
**13**	20.00		20.00		20.00		20.00		20.00		20.00		20.00		140.00	**1454.50**
**14**	20.00		20.00		20.00		20.00		20.00		20.00		20.00		140.00	**1594.50**
**15**	20.00		20.00		20.00		20.00		20.00		20.00		20.00		140.00	**1734.50**
**16**	20.00		20.00		20.00		20.00		20.00		20.00		20.00		140.00	**1874.50**
**17**			20.00				20.00				20.00				60.00	**1934.50**
**18**	20.00				20.00				20.00				20.00		80.00	**2014.50**
**19**			20.00				20.00				20.00				60.00	**2074.50**
**20**	20.00				20.00				20.00				20.00		80.00	**2154.50**
