# Supplementary material for: Tobacco treatment incorporating contingency management, nicotine replacement therapy, and behavioral counseling for pregnant women who use substances: a feasibility trial
Source: Front Psychiatry. 2023 Aug 16;14:1207955. doi: 10.3389/fpsyt.2023.1207955 (PMC10467262; doi:10.3389/fpsyt.2023.1207955)
Supplement: Supplementary Data Sheet 4 — Tobacco treatment manual. [file Data_Sheet_4.PDF]

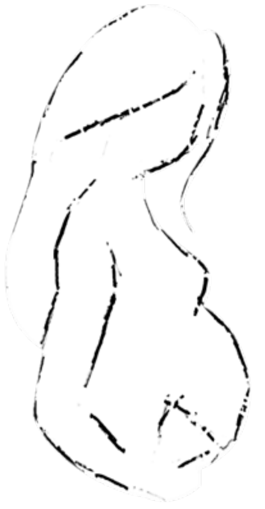

Incentives to Quit tobacco in Pregnancy

(IQuiP)

TOBACCO TREATMENT MANUAL

A guide for Counsellors

Contents

1 Overview ... 4
1.1 Rationale for counselling approach ... 4
1.2 The 3-Phases of counselling support ... 4
1.3 Assumptions ... 5
1.4 Trauma informed care in practice ... 5
1.5 Language ... 5
1.6 Counselling Delivery ... 7
1.6.1 Using this guide: ... 7
1.6.2 Historical Data ... 8
1.6.3 Number of sessions: ... 8
1.6.4 Delivery mode: ... 8
2 Phase 1 - Engaging women in the change process ... 9
2.1 Overall Aims: ... 9
2.2 Goals: ... 9
2.2.1 Establish rapport ... 9
2.2.2 Housekeeping ... 9
2.2.3 Relationship with tobacco ... 10
Smoking history ... 10
Barriers to addressing tobacco ... 10
2.2.4 Stigma Reduction ... 10
Recognise personal strengths ... 10
Identify positive health strategies ... 10
2.2.5 Tobacco addiction education ... 11
Understanding tobacco dependence ... 11
Effect of nicotine on the brain ... 12
Nicotine addiction cycles ... 12
Harm causing agents in cigarettes ... 13
2.2.6 Understanding dual dependency ... 13
3 Phase 2 - Guiding Cessation ... 16
3.1 Overall Aims: ... 16
3.2 Goals: ... 16
3.2.1 Increase motivation for behaviour change ... 16
Decisional balance ... 16
Reframing techniques ... 17
Motivational Question Guide ... 18
3.2.2 Identify social supports ... 19
3.2.3 Benefits of smoking cessation ... 19

Baby health benefits 19

Maternal health benefits 20

3.2.4 Preparing to stop smoking: 20

Reduce tobacco intake 20

Cigarette fading 21

Using NRT 21

Resisting the smoking urge 23

4 Phase 3 - Supporting smoking cessation & relapse prevention 24

4.1 Overall aims: 24

4.2 Goals: 24

4.2.1 Identify high-risk situations & solutions 24

Looking forward, looking back 24

Link emotions to cigarette-free solutions 24

4.2.2 Strategies to deal with stress 25

Relationship between stress and nicotine levels 25

Mindfulness strategies 25

Self-compassion 26

4.2.3 Lapse and relapse in high risk situations 27

Pathway of relapse 27

Dealing with cravings 28

Problem solving techniques 29

Conflict resolution 30

Grounding technique: 30

4.2.4 Ongoing support: 31

External quit smoking support providers 31

4.2.5 Promote long-term success: 31

Strategies for a healthy lifestyle 31

Sleep Improvement 32

Drug interactions with smoking cessation 33

4.2.6 Reward Success 34

5 References 35

6 Appendix 38

6.1 Session Guide 38

1 OVERVIEW

1.1 RATIONALE FOR COUNSELLING APPROACH

The counselling guide incorporated into the iQuiP smoking intervention has been adapted using two evidence-based interventions specifically targeted to pregnant women. The first is a comprehensive practice guide produced by the British Columbia Centre of Excellence for Women's Health titled *'Liberation! Helping women quit smoking: a brief tobacco-intervention guide'* (1). This evidence-based guide was produced to support service providers in the provision of cessation assistance to women. The second is *'A pragmatic guide for smoking cessation counselling for pregnant Aboriginal and Torres Strait Islander smokers'* (2) provided both cultural and technical understanding for the development of this manual.

This counselling approach is based on the recommendations of a review of the best-practice approaches to smoking cessation in pregnant women (3). It provides a tailored approach, specific to the needs of pregnant women who use substances, with a focus on women-centred care, stigma reduction, and attention to relapse prevention.

Motivational interviewing (MI) and Cognitive behavioural therapy (CBT) provide the basis for the content and delivery of the counselling. MI is a goal-orientated, client-centred style of counselling designed to engage clients and reduce ambivalence (4). It has been used to enhance treatment outcomes in smokers, including those who are pregnant (5, 6). MI focuses on collaboration with the client to enhance intrinsic or internal motivation and encourages client self-direction or autonomy to guide change. Techniques used in the guide include the exploration of mixed feelings about change, drawing out change talk and decisional balance.

CBT is an evidence-based talking therapy that works toward reframing maladaptive thought patterns and modifying the behaviours associated with them. It too has been associated with increased smoking abstinence in pregnant women in combination with pharmacotherapy (7, 8). CBT techniques include education around addiction and smoking, recognition of smoking triggers, identification of social supports and problem-solving strategies.

1.2 THE 3-PHASES OF COUNSELLING SUPPORT

A non-prescriptive approach to counselling support has been taken to encompass the individual needs and circumstances of our participants and the varying time they will spend enrolled in the study. Instead of a pre-determined number and structure of sessions, there is a recommended number to complete before birth and in the postnatal period. Additional sessions may be provided where necessary and less where time on-study does not permit.

The support will be broken into three, non-linear phases, allowing the flow of treatment to move backward and forward between the three, as required by the individual participant (see figure 1). The phases are:

1. Engaging women in the change process
2. Guiding smoking cessation
3. Supporting cessation and relapse prevention

Each phase has goals and strategies that are drawn from motivational interviewing and CBT to guide and assist women through this stage of significant behaviour change.

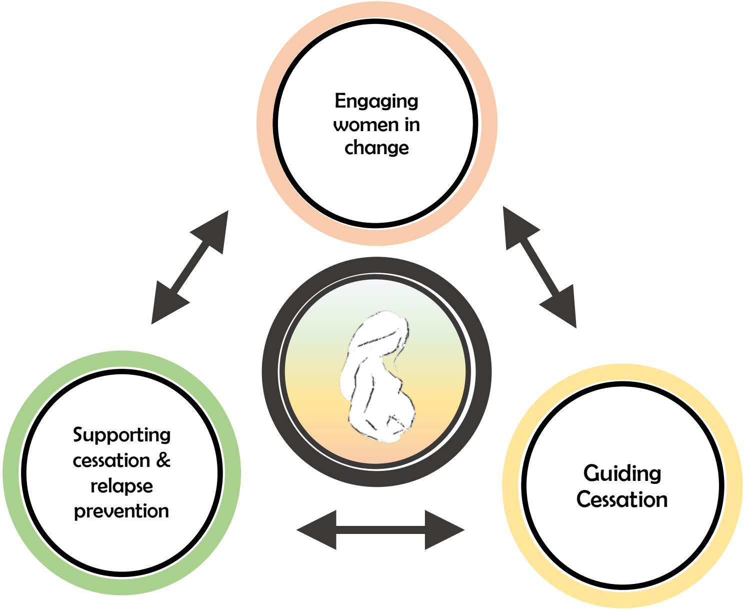

Figure 1: The three phases of counselling

1.3 Assumptions

The values and assumptions that are central to the counselling are identified as:

- Behavioural tobacco treatments for pregnant women have emphasised fetal health and 'giving up for baby', however, this does little to enhance the motivation required for long-term smoking abstinence (9). The cessation support used in this study is women centred and as such, is focused on participant's health both during pregnancy, postpartum and beyond.
- Research shows that a didactic approach to treatment can hinder motivation to stop smoking in pregnant women (10). Counsellors must work collaboratively and sensitively with women throughout the counselling process. Expertise is shared; participants are experts in their own lives while counsellors can provide the knowledge relevant to cessation.
- The participant's physical and emotional safety is paramount. Counsellors will employ a trauma informed approach with a focus on making women feel safe. Doing so can help to reduce potential negative emotional responses and subsequent triggers or urges to smoke.
- Pregnancy is thought of as a time of high motivation to stop smoking and can be described as a 'teachable moment' for smoking treatment. The reality in complex clients is that often, there has often been no deliberate decision to become pregnant, substance use treatment is prioritised and partners and other household members smoke, so motivation to quit can remain low.

1.4 Trauma informed care in practice

Trauma informed practice is an approach to behavioural treatment that acknowledges the prevalence of trauma and how it can affect a person's life. An informed practice or service delivery recognises the impact that trauma can have on child development and adult mental health and, importantly, provides a framework for being able to minimise these effects without causing additional trauma.

Counsellors should be aware of the links between past traumatic experience and current problems of living. They need to be mindful that pervasive impacts of trauma may include the way participants approach potentially helpful relationships, and therefore be mindful to avoid re-traumatisation. They need to be

committed to, and act upon, the core principles of safety, trustworthiness, choice, collaboration and empowerment, and be mindful that positive relational experiences provide enormous potential for recovery.

In the context of the current treatment, it is expected that counsellors will provide a trauma-informed approach. While exposed trauma will not be treated directly, it must be understood that it will underpin the presentations of many research participants. Additionally, the potential exists for trauma to still exist in their lives, so counsellors should be informed and sensitive to trauma-related issues.

Lastly, this research provides the opportunity to maintain some women in counselling for an extended period. All counselling, research and antenatal staff should maintain a collaborative approach to ensure that they receive continuous care, as integrated care reduces risk of negative experiences, active re-traumatisation and compounding unrecognised trauma of participants.

1.5 Language

Stigma is defined as a mark of disgrace that is associated with a circumstance, quality or person (11). Addiction is one of the most stigmatised of health conditions and is influenced by two factors: cause and control. The less that people believe a condition is caused by or is under the control of themselves or an individual, the less stigma is associated with it (12). The use of language can influence how those with substance use problems see themselves, as well as our own judgement and treatment of them.

The following table[1] is designed to increase awareness around the use of language and provide some alternatives to help reduce the stigma associated with smoking and substance use in pregnancy. The basic premise is to use language that promotes empowerment and fosters hope. This can be achieved by the consistent use of "person first" language and avoiding terms that induce bias, stigma and self-criticism.

Avoid saying…	Alternative(s)	Why?
Words that describe the person e.g. 'she is a smoker' or 'she is an addict'	Words that describe behaviours e.g. 'she smokes' or 'she is using a substance'	This reflects the behaviour and not who the person is.
Drug use; drug abuse	Substance use or substance misuse	This describes the behaviour in a neutral way. The word 'drug' can have adverse connotations.
Quit smoking	Stop smoking or reduce to zero cigarettes<br>Using nicotine products instead of smoking	This describes the behaviour in a neutral way. 'Quit' can have negative connotations for some e.g. as it can imply pressure or feelings of loss
Slip up; Relapse	Had a smoke or a couple of cigarettes	These are less critical and remove implications of failure or of wrong doing.
Disease; Illness	Condition	Diseases are often avoided or feared. A condition is something that can be managed.
Control	Manage	Control suggests that a woman can dictate her substance use behaviour. Trying to stay in control can lead to feelings of failure and guilt when goal attainment does not succeed.

[1] Adapted from 09 Tip Sheet – Quitline Victoria (ask AB)

Avoid saying…	Alternative(s)	Why?
You should/should not; must/must not; can/cannot or you have to…	Have you considered…?<br>What would it be like if…?<br>Are there other things you could try…?	Using open-ended questions instead of dictating direction is helpful for this population of women who often focus on "should haves" and "failures."<br>Asking questions or providing suggestions gives options and acknowledges a woman's choice in determining her own health.
When baby is home…	After delivery…	New-borns from this population are at increased risk of admission to a neonatal intensive care or being assumed into care (13).
Give up for your baby…<br>The health benefits to your baby…	Your health and wellbeing	It is important that attention is paid to woman's health, not just baby. The need to stop smoking can be extrinsically motivated by pregnancy. Long term change should be motivated by intrinsic (concern-for-self) motivation (9).
Counselling	Cessation support or support<br>Support call	The term counselling has negative connotations for some people. The thought of bringing up past trauma (as is the case in many instances of counselling) can lead to avoidance. The term support removes these associations and is suitable for smoking cessation treatment.

1.6 Counselling Delivery

The counselling support provided will form one part of the comprehensive smoking treatment offered by the iQuiP study. It is designed to support participants through smoking reduction and/or cessation and compliment the contingency management and pharmacotherapy components of the intervention.

1.6.1 Using this guide:

The following guide contains the appropriate resources required to move women through the smoking cessation process. Each phase has treatment aims and goals, with strategies provided to achieve these.

A flexible delivery approach allows each session to be personalised. Collaboration and letting participants direct the counselling process, will guide which strategies are used to meet their individual needs. There is a large amount of content in each phase of the counselling support, it is not expected that this would all be covered within a single session.

A separate session guide acts as a record of each participants counselling involvement. It contains standard information to be collected for each session and a list of themes contained in the three phases of treatment. As a theme is covered in session, it can be checked off. Space for note taking is also provided.

A participant workbook and viewing resource containing copies of information and illustrations used in the guide is included. This may be used for visual support during videoconference sessions or used to make notes if desired.

A clinical summary from each session must be written into the participant's clinical health records (CHIME & CHOC).

1.6.2 Historical Data

To prevent burdening participants with repetitive questioning, the information collected during the baseline research interview is available to counselling clinicians. This contains demographics, tobacco smoking history, current smoking status and partner/household smoking, current substance use, smoking dependence (Fagerström Test for Nicotine Dependence; FTND), quit attempt history, mental health assessment (Patient Health Questionnaire; PHQ-12 & Generalised Anxiety Disorder; GAD-7), alcohol use (Alcohol Use Disorders Identification Test – Consumption; AUDIT-C), substance use (Australian Treatment Outcome Profile; ATOP), and childhood trauma (Childhood Trauma Questionnaire; CTQ).

This de-identified information is available in report format on REDCap. This customised report can be retrieved from the Data Exports, Reports tab and by selecting the 'Counselling Report'. A login is required; please contact the research coordinator Mel.Jackson@hnehealth.nsw.gov.au to organise this if necessary.

1.6.3 Number of sessions:

Ideally, up to 6 sessions of support should be provided; four before birth and two in the postpartum period, to assist relapse prevention. This will vary depending on women's gestational age at recruitment and their individual needs. Additional sessions may be delivered if needed or requested.

1.6.4 Delivery mode:

Delivery shall be by videoconference using Telehealth (preferred) or over the telephone if necessary. Telehealth allows for visual communication between clinician and participant while removing the logistical barriers often associated with lack of treatment, or missed attendance, in this group of women.

It may be helpful to give an explanation of the benefits of using telehealth for counselling sessions to participants. These include:

- more convenient; no need to leave home, no travel time
- more cost effective
- more flexible for other children
- reduces need for childminding
- flexible appointment times

Telehealth does, however, require commitment from both parties. The counsellor and participant must commit to an agreed appointment time and both will need to dial into the telehealth portal to access the service. It is important to explain that the participant needs to set aside time for the counselling session. This may mean, for example, getting assistance with occupying other children, finding a quiet space away from partners or other household members, or turning off music or other distractions.

Telehealth is not without technical difficulties, and it is important to explain that dropouts or bad connections may occur. In the case of a dropout, it is up to the counsellor to contact the participant as soon as practical so that the session can be completed over the telephone.

Telehealth requires a mobile application to be available on participant's telephone or other internet enabled device with a camera. This should be downloaded by the research team as each participant joins the study but check that the participant is aware of the app and it's use before making the first appointment.

In exceptional circumstances or in instances where it is expressed or clinically indicated, face-to-face counselling sessions should be made available to participants. An example may be where evidence of family violence exists and a counselling session taking place in the home may increase the risk of violence.

2 PHASE 1 - ENGAGING WOMEN IN THE CHANGE PROCESS

Traditional smoking cessation programs have low levels of success with complicated client populations (14). These programs are often targeted at those already motivated to quit and fail to offer the level of support required to treat complex needs, making them ineffective for those who use substances and smoke concurrently.

Other barriers to cessation exist in this population of smokers. These include high levels of nicotine dependence and high rates of partner and household smoking; the use of cigarettes for stress relief; social and cultural smoking norms; lack of cessation support; coping with dual dependencies; poor mental health and socioeconomic disadvantage (14-16). It is important that clinicians treating smoking understand these and use a tailored approach that can help overcome such barriers and encourage behaviour change for this group.

The initial component of this smoking treatment is about engagement in the behaviour change process. It is imperative that rapport is established prior to incorporating other aspects of the counselling designed to increase her readiness to quit.

2.1 OVERALL AIMS:

i. Set the tone for the counselling process i.e. non-judgemental, non-directive
ii. Develop women's self-efficacy to reduce or stop smoking.
iii. Work collaboratively to begin the smoking reduction or cessation process.
iv. Direct the change focus away from baby's health and onto the woman and her short- and long-term health.

2.2 GOALS:

- Establish rapport
- Housekeeping
- Explore relationship with tobacco
- Reduce shame and guilt about smoking
- Provide education on tobacco dependence
- Understanding dual dependency

2.2.1 Establish rapport

- Make Introductions
- Praise the decision to address smoking

2.2.2 Housekeeping

- Discuss participant rights and responsibilities regarding counselling. These should reflect the mode of counselling delivery and include reminders that smoking and substance use are not permitted during the videoconference call, even though the participant may be within the confines of their own home.
- Discuss privacy issues
- Explain the study counselling process, including suggested number of sessions pre- and post-delivery, the length of each session and what will happen in each session, including stop smoking plans and dates.
- Advise that more sessions are available if required.
- Explain Telehealth teleconferencing, including setting appointment times, dial-in procedures, data use, problems that can occur e.g. bad connections
- A backup plan should a video call not be available/accessible e.g. call or text to organise phone session.

2.2.3 Relationship with tobacco

Smoking history
General discussion around smoking context and history can help rapport building. General information about the woman's background and smoking history has been collected during the baseline research interview and is available on the REDCap database (see section 1.6.2).

- Explore what motivated the decision to participate in research to give up smoking.
- Discuss the positives and negatives of smoking
- Discuss any previous attempts to stop tobacco and/or other substances, and what strategies the participant used.
- Make links to the psychological and situational cues that maintain tobacco use e.g. social smoking, triggers such as coffee or alcohol, stress, substance use, stress
- Talk over any other aspects of smoking that may have been raised

Barriers to addressing tobacco
There are many different reasons why women smoke, and these can be a barrier to their wanting to stop. Common reasons include weight gain, withdrawal symptoms such as irritability or moodiness and social norms around smoking (e.g. smoking with partner, friends etc.).

- Discuss as many reasons as possible that the participant can identify that might prevent her from wanting or trying to stop smoking.
- Reframe (see phase 2 – **Encourage talk about smoking related changes**), provide information and/or devise strategies that can help to mitigate these concerns.
- Use the Decisional balance technique (refer 3.2.1 – **Increase motivation to change**)

2.2.4 Stigma Reduction

Women who use substances, including tobacco, during pregnancy face substantial stigma that can have a negative impact on their own mental health and the health of their baby. It is important to normalise addiction and identify individual strengths or other ways to support good health. This will help reduce a woman's guilt around her substance use and smoking.

Recognise personal strengths
Identify current strengths and evidence of resourcefulness that may contribute to the development of self-efficacy and feel more comfortable about themselves, their pregnancy and baby. These might include:

- Agreeing to participate in the study. This shows strength and a will to want to stop smoking.
- Any previous attempts to reduce or stop smoking or substances. This provides opportunities for pre-learning.
- Attending antenatal clinic appointments. This shows resourcefulness.

Identify positive health strategies
Encourage women to think about other things that they can do for a healthy pregnancy and caring for their unborn child. These can include:

- Getting enough sleep (refer to 4.2.5- **Sleep improvement**)
- Eating well and regularly (refer to 4.2.5 – **Strategies for a healthy lifestyle**)
- Learning how to better deal with stress (refer to 4.2.2 - **Mindfulness and relaxation strategies**)
- Increasing activity levels, exercising
- Going for regular prenatal health check-ups
- Staying emotionally healthy (refer to 4.2.5 - **Strategies for a healthy lifestyle**)

2.2.5 Tobacco addiction education

The following is some general information about tobacco smoking, addiction and other substance use. The participant may already understand some of these or may not be interested in learning. Ask what she has heard about and/or what she would like to know more about:

Understanding tobacco dependence

Education around nicotine addiction and how it forms can be effective in normalising smoking addiction and helping participants to begin making changes to their smoking behaviours.

Physical Dependence

- This is the addiction, caused by repeated exposure to nicotine through tobacco.
- The body needs nicotine to function normally. Without it, the body goes into nicotine withdrawal
- Withdrawal symptoms can include:
    - Cravings or urges to smoke
    - Irritability, anger, anxiety, sadness, depression
    - Restlessness or difficulty concentrating
    - Sleeping difficulties or disturbances
    - Increase in hunger
- Providing the body with nicotine can reduce these symptoms e.g. NRT

Psychological Dependence

- This is the habit of smoking, triggered by the cues that have been repeatedly linked to smoking
- These cues can be:
    - Situational e.g. smoking with coffee or alcohol
    - Emotional e.g. smoking when feeling stressed
    - Visual e.g. seeing someone else smoking
    - Physical e.g. smelling cigarette smoke or having nothing to do with your hands
- Cues create urges to smoke
- Identifying the individual cues that trigger smoking is the first step in breaking the tobacco habit

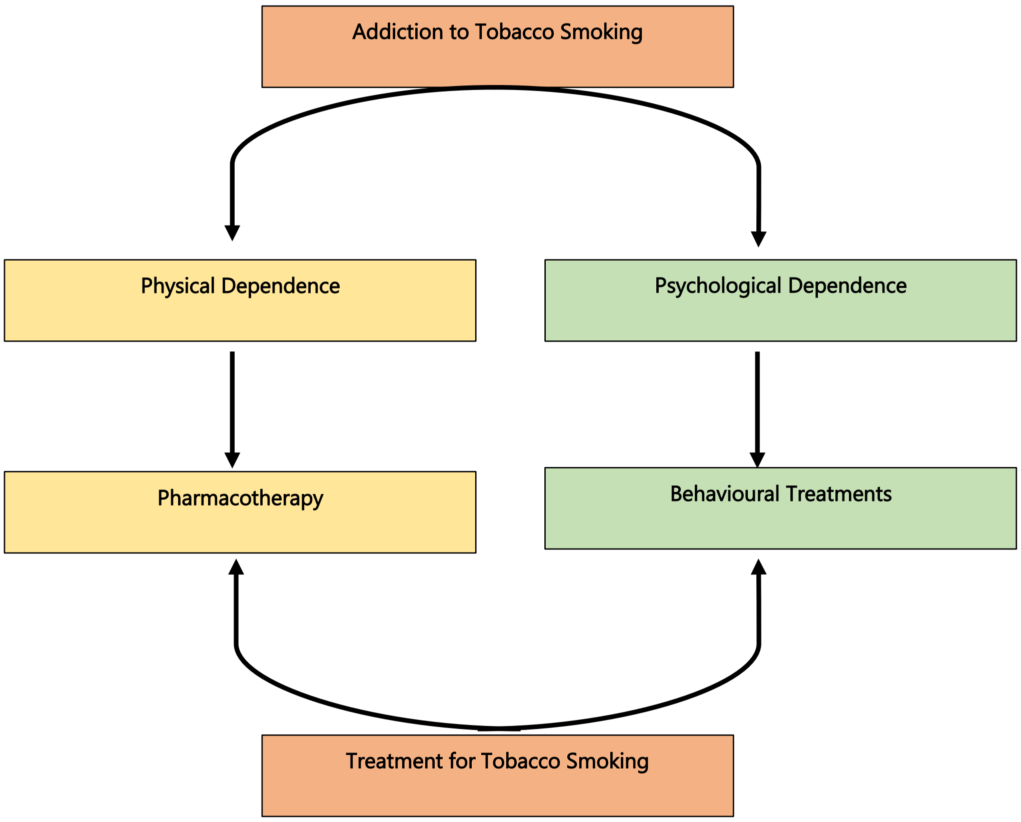

Effect of nicotine on the brain

Nicotine is the active ingredient in cigarettes that causes dependence and addiction. It is also a stimulant that affects memory and concentration, increases heart rate and reduces appetite. Nicotine enters the brain via the brain stem, attaching itself to nicotine receptors that activate the brain's reward centre. Here it stimulates large amounts of the 'feel good' hormone dopamine, a chemical that helps to reinforce behaviours that are pleasurable.

Nicotine and the release of dopamine also trigger changes in the pre-frontal cortex, an area associated with judgement, memory and logic, by changing the *liking* of something to a *wanting*. This makes a smoker motivated to seek out more nicotine.

Generally, nicotine levels peak around 10 minutes after inhalation, but dissipate quickly, creating the continual need to smoking throughout the day.

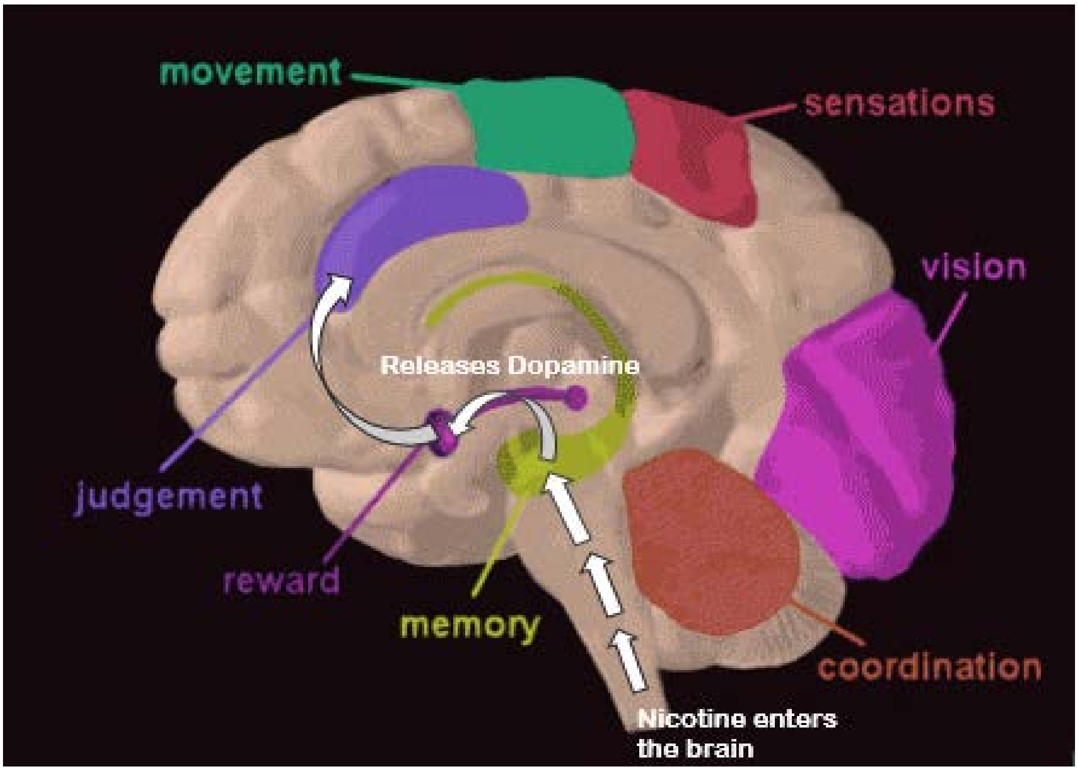

Nicotine addiction cycles

The figure below illustrates the typical nicotine withdrawal that a tobacco smoker experiences during the day. Each peak represents peak nicotine after a cigarette while each trough shows the rapid drop of nicotine (this takes approx. 30-40 minutes). The first cigarette of the day has the most pronounced effect, with peaks outside the upper tolerance level indicating pleasure and lower tolerance level indicating abstinence and withdrawal symptoms. The nicotine tolerance level (between the two horizontal lines) represents the area that a smoker feels comfortable without experiencing symptoms of either (17).

Nicotine inhaled from cigarettes enters the blood stream and rapidly reaches the brain before being reinforced within seconds. This, and the addition of chemical additives designed specifically to increase addictiveness (17) mean that addiction to nicotine and cigarettes occurs quickly.

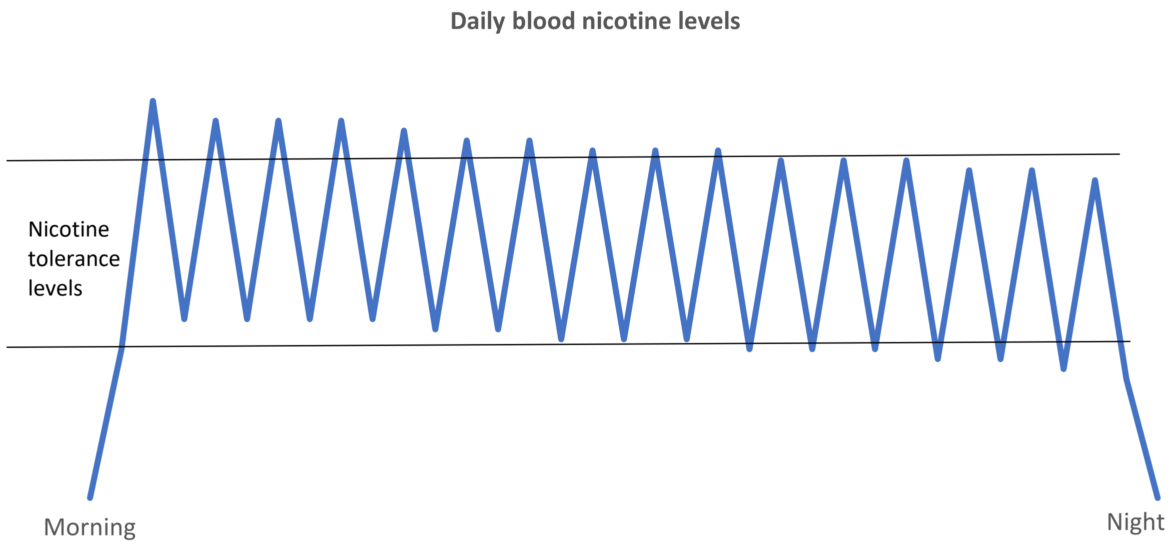

Harm causing agents in cigarettes
Cigarettes contain approx. 7000 harmful chemicals that are ingested into the blood stream with every cigarette smoked. Some of the more recognisable chemcials are listed in the table below.

It should be highlighted that nicotine is only one of many harmful agents found in cigarettes, but the only one contained in NRT. *Refer to the participant resource guide for pictorial view of this information.*

**Chemcial**	**Commonly found in**
Nicotine	Insecticides
Acetone	Nail polish remover
Butane	Lighter fluid
Petrol	Car motors
Paint	Household paint
Cadium	Batteries
Arsenic	Poison
Acetic acid	Vinegar
Formaldehyde	Embalming fluid
Carbon Monoxide	Car exhaust
Toluene	Industrial Solvent
Stearic Acid	Candle wax
Hexamine	Fire lighters
Ammonia	Toilet cleaner
Methane	Decay of organic waste

2.2.6 Understanding dual dependency

Concomitant tobacco and drug use
The following table provides information on the interactions between tobacco smoking and other drug use.

Smoking with other Drugs [2]	
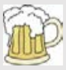<br>**Alcohol**	➢ Nicotine and alcohol each enhance the enjoyment of the other through their common action in the reward pathway, where they both trigger the release of dopamine.<br>➢ Drinking alcohol increases the urge to smoke partly due to the disinhibiting effects of alcohol and conditioned association of the two behaviours<br>➢ Smoking increase the urge to drink and is a risk factor for relapse to alcohol after alcohol treatment.<br>➢ Smoking reduces the sedative and cognitive effects of alcohol and reduces the severity of alcohol withdrawal.
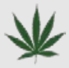<br>**Cannabis**	➢ Frequently smoked with tobacco<br>➢ Two out of three cannabis smokers add tobacco to their mix, leading to higher levels of nicotine dependence and continued cigarette smoking<br>➢ Quitting both together is recommended as continuing use of cannabis can make quitting tobacco harder<br>➢ Cannabis and tobacco have similar withdrawal syndromes and the combined symptoms may be more severe<br>➢ Strategies used for tobacco cessation (including NRT) can also help reduce or stop cannabis
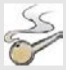<br>**Stimulants**	➢ More than 80% of people who use stimulants smoke tobacco<br>➢ Stimulant administration increases tobacco smoking and nicotine use increases the severity of stimulant use<br>➢ Nicotine withdrawal (quitting smoking) is associated with reduced cravings for stimulants<br>➢ Quitting smoking during stimulant treatment improves smoking abstinence and does not undermine stimulant outcomes
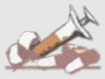<br>**Opiates**	➢ People who use opiates and opioid agonists (methadone, buprenorphine) have the highest smoking rates (>85% smoke).<br>➢ Methadone may increase the reinforcing effects of cigarettes. People who use methadone generally smoke more heavily in the four hours after each dose.<br>➢ Nicotine is known to weaken some side-effects of methadone such as sedation and reduced attention.

Stopping two substances together

It is not uncommon to have two addictions at the one time; over two-thirds of people in Australia with an alcohol or other drug disorder also smoke (18). While smoking has traditionally been given lower priority in treatment settings, research suggests that concurrent smoking cessation can improve alcohol and other drug (AOD) treatment outcomes (19). The following table cites common myths around concurrent cessation and provides supporting evidence for its benefits.

[2] Adapted from Mendelsohn C. & Wodak A, (2016), Smoking cessation in people with alcohol and other drug problems. Australian Family Physician 45(8)

Stopping two substances together	
**Common Assumptions**	➢ People who smoke and use other substances are not able to stop, so smoking treatments are a waste of time and resources<br>➢ People who smoke who use other substances don't want to stop smoking<br>➢ Smoking is less dangerous or less important than other substances of dependence<br>➢ Stopping two substances at once is too difficult and will impair the chance of abstaining from either substance
**Evidence for Concurrent Treatment**	➢ People seeking treatment for substance use report a desire to stop smoking tobacco at similar rates to the general population of smokers (20)<br>➢ People treated for substance use and smoking simultaneously are 4 times more likely to achieve long-term abstinence from alcohol and other drugs than those who don't receive smoking treatment (19)<br>➢ Those who gave-up smoking during their first year of AOD treatment were more likely to be abstinent from alcohol &/or other drugs after 9 years than those who didn't (21)<br>➢ Smoking can cause a relapse to alcohol and/or other drugs (22)<br>➢ Smoking cessation during or after AOD treatment can lead to better outcomes for abstinence from other drugs (21)

3 PHASE 2 - GUIDING CESSATION

3.1 OVERALL AIMS:

- Build self-efficacy
- Encourage reduction and cessation

3.2 GOALS:

- Increase motivation for behaviour change
- Identify social supports
- Provide education on smoking cessation
- Preparing to stop smoking
- Mental health, medication and smoking cessation

3.2.1 Increase motivation for behaviour change

Some participants will need encouragement to contemplate life without cigarettes. Motivation to change tobacco smoking behaviour comes from within. It is important to encourage talk about change, without demanding change. A participant, hearing themselves speak, can reinforce their desire to stop smoking and increase the belief in themselves to do so.

Decisional balance

The decisional balance can help to explore the pros associated with quitting and the cons of continuing to smoke tobacco without influencing the direction of behaviour change. It aims to uncover the participant's ambivalence around smoking and creates an opportunity for them to hear themselves speak about what is for them, the negative aspects of smoking.

	**Benefits / Pros of stopping**	**Costs / Cons of smoking**
**No Change in smoking**		
**Change in smoking**		

Reframing techniques

Reframing is a way of changing the meaning, emotional context or viewpoint of a situation by putting it into another frame that more effectively fits the facts of the same situation. It challenges assumptions and can be used to create motivation.

The following reframing examples[3] challenge some of the more common feelings that can occur when trying to stop smoking.

Common thoughts	Suggested Responses
Smoking is the least of my problems	Often tackling one problem can benefit other areas or problems. For example, those who stop smoking are more likely to stay off other substances. Also, staying off cigarettes can save money and help relieve financial stress
It is too difficult to quit two substances at once	While difficult, people report that it is easier to use no substances than stop one at a time. This is because smoking is often linked to the other substance use e.g. drinking alcohol and smoking, spinning cannabis with tobacco. Research has found that people who stop smoking are more likely to stay off other substances (21). Smoking can also increase the likelihood or relapsing to other substances (22).
I need cigarettes to help me cope or relieve my stress	People who smoke tend to believe that smoking relieves anxiety and stress yet stopping smoking can help people cope better. Part of the stress felt is the body going into nicotine withdrawal between cigarettes (refer to 2.2.5 *Nicotine Addiction Cycles*). Most people will find that stress reduces, and they can cope better within 6-weeks of giving up. Practicing mindfulness may be helpful in this case.
Smoking is the lesser of two evils	The problem about smoking is that the many physical harms are not experienced until much later. The harm experienced by alcohol and other substances might be felt much sooner e.g. overdose, criminal conviction
I can't give up everything…smoking is my only remaining vice	Some people believe that smoking gives pleasure, although it is more likely to be the circumstances around smoking such as the social aspects, or the relaxation from taking time out to have a cigarette. Everyone deserves activities that provide pleasure, so smoke-free alternatives should be discussed.
I don't want to use NRT as I will still be addicted to nicotine.	It is the inhalation of smoke and the chemicals within cigarettes that causes significant health damage, not nicotine. It is rare for people to become addicted to nicotine replacement products, but for those that do, it is less expensive and much less harmful than tobacco smoking.
Smoking is just a habit, a way of life	Smoking is both a physical addiction and a psychological dependence. Breaking the habit of smoking is critical to stopping, however, it is also important to treat the addiction and symptoms of withdrawal.
No one has bothered about my smoking before	It was not uncommon for tobacco smoking to be viewed as the lesser of two evils when treating substance use or mental health. It was thought that stopping tobacco use would cause people to return to other substances. We now know that treating smoking can improve substance use treatment, so there is more focus on it.
It's too late, the damage from smoking & other drugs is already done	It's never too late to stop smoking and the benefits to health are immediate (refer to 3.2.3 - *Health Changes after smoking cessation*). Most people who stop smoking will feel better physically and mentally in the long run.
It's not important or worth the hassle to stop smoking	It does seem like hard work but stopping makes such a positive difference to the health of a woman, and assists during pregnancy, that any attempt to reduce smoking is worth it.

[3] Adapted from Quitline Victoria, see AB re reference

Common thoughts	Suggested Responses
I don't care if it kills me, you have to die of something	This may be true, but death from smoking is preventable and death is likely to occur much earlier than it should. Everyone deserves the financial and health benefits of not smoking,
I'm so bored now and I always smoke when I'm bored	It is common to feel bored when cutting down or stopping smoking for good. Therefore it is a good idea to identify things that you enjoy that can make you feel less bored. (Refer to 3.2.4 *DEEDS Strategy*)
It's all too much and too hard	Yes it is difficult because you are making changes to smoking, and nicotine is an addictive and powerful drug. Also remember that withdrawal symptoms and cravings get a little easier to handle every day, until they eventually stop for good. When feeling that it is all too hard, consider using NRT, speaking to your social supports. It might also help to think about the financial incentives and health benefits gained from not smoking.
It's not the same (without cigarettes)	There is adjustment associated with stopping any habit. It might feel like there is more time in the day, or you have a sense of loneliness. It is common to feel 'different' without the cigarettes that have previously taken up a large part of life.
Smoking is my best friend	Smoking can feel like a friend, giving support when you need it. It is normal to have feelings of loss when you stop, with the void created by not smoking often interpreted as loneliness. It is worth wondering whether a friend would cause as much harm, and cost as much money, as cigarettes do.
I've tried to quit before and I failed	It is common to have many attempts at stopping before being successful. Each of these attempts is a valuable lesson about relapse triggers, and knowing these can make the next attempt more successful. Combining strategies, like using NRT with counselling support and financial incentives can help.

Motivational Question Guide

The following questions are a motivational technique that can help create conversations about smoking cessation. An exploration of the answers will identify strengths and skills as well as barriers and motivation blockers. Affirmation and problem solving will help to increase motivation.

Questions for Change
➢ "What would change in your life if you stopped using tobacco?"
➢ "If you make no changes around tobacco smoking, how do you see yourself in 5 years' time?
➢ "If you did stop smoking cigarettes, what would be the thing you would most look forward to?"
➢ "What are you concerns around making changes to your tobacco smoking?"
**Questions to build confidence**
➢ "On a scale of 1-10, how confident are you that you can stop tobacco smoking, where 1 is not confident and 10 is very confident?"
➢ "Why did you give yourself that number and not a lower one?"
➢ "What would it take to move the number up one?"
➢ "Is there anyone in your family or circle of friends that could help you make changes to your smoking?"
➢ "Thinking about a time when you made changes to your substance use, is there things that you learnt that could help you now stop smoking?"

➢ "What would a close friend describe as your strengths that would help you to stop smoking?"

3.2.2 Identify social supports

Evidence suggests that social networks with high smoking prevalence and smoking acceptability become a large barrier to smoking cessation (14). Spending less time with these high-risk networks and more time with support networks with fewer smoking influences in the early stages of a quit attempt can lead to higher rates of success (23).

- Are there friends and/or family members who are aware of the participant wanting to stop smoking that could provide support during the quitting process? Discuss how and when these can be helpful.
- Are there recreational interests or hobbies that could help with distraction and keep participant occupied during the quitting process?

3.2.3 Benefits of smoking cessation

Although it is general knowledge that smoking during pregnancy is detrimental to the developing baby, it may be useful to explain in more detail how smoking causes these effects and the benefits of stopping. Always ask permission before providing such information and don't assume that she doesn't already know or wants to know this information.

Baby health benefits

**Smoking and Pregnancy**
➢ Optimal fetal development requires a healthy placenta joining a baby to its mother in the womb.<br>➢ The placenta is vital for supplying all the oxygen, nutrition and life support from the mother's blood to the baby
**The effects of smoking on a mother's body:**
➢ Nicotine tightens the blood vessels in the uterus, reducing blood flow to the placenta and baby<br>➢ Smoking produces carbon monoxide in the mother's blood stream, making less oxygen available to the placenta for baby<br>➢ The harmful chemicals contained within cigarettes are absorbed into the mother's blood stream and passed directly into the placenta and onto baby
**Smoking cessation reduces the risk of:**
➢ Miscarriage<br>➢ Low birth weight<br>➢ Pre-term or premature birth<br>➢ Poor lung development (leading to ongoing respiratory problems)<br>➢ Birth defects including cleft palate & heart defects<br>➢ Weak immune system<br>➢ Need for neonatal intensive care<br>➢ SIDS<br>➢ Future behavioural problems (including ADHD, substance use)<br>➢ Future tobacco smoking
**When is the best time to stop?**

- ➢ Stopping at any time during pregnancy is beneficial, although cessation early in the pregnancy will always provide the best start in life for baby.
- ➢ Stopping before 20 weeks will provide the best results for birthweight, although smoking cessation in the second half of pregnancy will also result in birthweight improvements (24)

Maternal health benefits
It is never too late to give-up smoking – the health benefits are immediate and continue to grow over time. Not all benefits are visible or noticeable, so it can be useful to highlight the changes that will take place in the body over time.

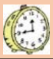	**Health changes after smoking cessation**
**After 30 minutes**	Blood pressure, heart rate, temperature of hands & feet become normal
**After 12 hours**	Carbon monoxide and oxygen levels in the blood return to normal
**After 48 hours**	Sense of taste and smell start to return to normal levels
**After 72 hours**	Bronchial tubes relax, and breathing is easier
**After 1 week**	Nicotine is flushed from your body
**After 2 weeks**	Circulation, breathing, and lung function improve
**After 1 month**	Coughing, sinus congestion and shortness of breath decrease
**After 2 years**	Risk of heart attack drops to that of a woman who has never smoked
**After 5 years**	Risk of stroke drops to normal; risk of lung cancer decreases by half
**After 10 years**	Risk of most types of cancer drops to normal
**After 20 years**	Risk of dying from smoking-related causes is similar to women who have never smoked

3.2.4 Preparing to stop smoking:

Participants will be allowed to stop smoking abruptly or reducing the amount they smoke over 1-4 weeks before stopping completely. Evidence suggests that neither option offers more effective results over the other, regardless of whether other cessation aids are used or not (25). One benefit of reducing nicotine first is that it can remove the pressure of stopping, however, a target cessation date should be set and adhered to for the most effective results (26).

Reduce tobacco intake
It should be advised that cutting down can take considerable discipline to adhere to. The following are suggestions that can assist in the reduction of tobacco prior to stopping.

**Tips to reduce tobacco**
➢ Use NRT; the slow release of nicotine from a patch will reduce the body's need for nicotine from cigarettes
➢ Get into the habit of only smoking outside the house and/or not in the car
➢ Try only smoking part of a cigarette and throwing the rest away

➢	Use the cigarette fading technique (Refer to 3.2.4 – *Cigarette Fading*)
➢	Wherever possible, avoid those situations closely associated with smoking
➢	Take up new activities that distract and reduce available smoking time

Cigarette fading

The following strategy is designed to make a smoker more aware of their smoking and systematically reduce the number of cigarettes that they smoke in a day.

**Cigarette Fading**	
1	Determine a number of cigarettes to smoke on Day 1. This should be at the low-end of the range smoked per day e.g. 10 if 10-15 is the average number of cigarettes smoked
2	Have only this number of cigarettes available. Hide all others e.g. give them to a non-smoking friend; put them in the freezer; have a neighbour hide them etc.
3	Take notice of the length of time in between each cigarette during the day.
4	If you achieve your smoking target, set a new, lower target for the following day. Make sure you gradually increase the amount of time between each cigarette
5	If a target is not achieved, try other strategies to reduce the number of cigarettes smoked (Refer to 3.2.4 – *Tips to reduce tobacco*).
6	If still unable to meet the reduced target, stick with the original target number of cigarettes (e.g. 10), be mindful of your smoking and try implementing other strategies to reduce smoking.

Using NRT

The use of NRT as a reduction strategy has been shown to effectively decrease the number of cigarettes consumed per day and, at the same time, increase motivation to quit in general populations (27). Low uptake of NRT by pregnant women in Australia has been noted (28) despite evidence suggesting that it is safe to use during pregnancy (28).

The effectiveness of NRT during pregnancy has not been well documented in clinical trials. This is likely caused by the trialling of inadequate NRT dosages that fail to address the increased metabolism of nicotine during pregnancy (29). During pregnancy, the increase in levels of oestrogen and progesterone hormones, cause the body to clear nicotine at a rate much faster than in non-pregnant women (30). This increase in metabolism requires higher levels of NRT to adequately treat withdrawal symptoms and manage cravings, especially in women highly dependent on nicotine (31).

The following table describes nicotine and NRT in the context of pregnancy and explains its benefits in the cessation process:

Nicotine Replacement Therapy

Nicotine

- Nicotine is the main addictive ingredient, but only one of the approx. 7000 chemicals that are contained in cigarettes (refer 2.2.5 – *Harm causing agents in cigarettes)*
- Nicotine CAN cause some harm to the developing fetus and pregnant mother (based on animal studies), but is not considered to be as potent or harmful as other toxins caused by smoking, such as carbon monoxide, tar, lead and arsenic (31).
- Pregnant women metabolise nicotine at a higher rate than non-pregnant women (32), often hampering efforts to reduce or stop smoking.
- The benefit to mother and baby of reducing exposure to such harmful chemicals far outweighs the risk incurred by nicotine, so any treatment that can assist with this is beneficial.

Nicotine Replacement Therapy

- NRT is a medication that reduces nicotine withdrawal symptoms while the body adapts to a lack of nicotine. It also helps to stop or reduce smoking
- NRT provides a dose of nicotine without exposing the user to the other harmful chemicals
- The level of nicotine absorbed from NRT is lower and absorbed more slowly than that from cigarettes. For this reason, NRT is much less addictive than cigarettes (31).
- It comes in slow release forms (patches) and fast release forms (gum, lozenge, mouth spray and inhaler)
- Pregnant women need higher doses of NRT to reduce cravings and manage withdrawal symptoms than they would if they weren't pregnant.
- Using a combination of slow and fast release forms of NRT is most effective (33).
- Depending on nicotine dependence, a combination approach should be used, with the recommended dosage being as much NRT as is needed to overcome withdrawal symptoms and cravings to smoke.

Benefits of Nicotine Replacement Therapy

- Increases chances of having a healthy baby
- Much safer than smoking tobacco
- Decreases symptoms of nicotine withdrawal
- Increases the likelihood of smoking abstinence if used properly, for long enough and in high enough doses
- Helps to prevent potential weight gain
- Cheaper than smoking
- Multiple product options mean that NRT can be individualised to suit different people
- Can quickly and effectively help in a stressful or challenging time (when smoking would normally have occurred)
- Can be used to help reduce cigarette intake

Side-effects of NRT

Any side-effects are generally mild, but should they become troublesome, a change in product type or brand is advised. More serious symptoms should be referred to a GP.

- Skin irritations (patches)
- Irritation of throat (mouth spray, gum)
- Vivid dreams
- Difficulty sleeping
- Nausea
- Dizziness
- Upset stomach

Resisting the smoking urge

An effective technique when giving up smoking is the advance planning of ways to resist urges to smoke that occur as part of the withdrawal process. The following provides multiple suggestions and strategies, using the acronym, DEEDS, to allow easy recall.

**The DEEDS Strategy**	
**Delay**	➢ Delay smoking for 15 minutes<br>➢ Delaying gets easier with practice, you can delay for longer periods of time<br>➢ Delaying means smoking less at the end of the day<br>➢ Strategies to delay:<br>– Delay first cigarette of the day e.g. until after breakfast<br>– Set smoke free hours e.g. no smoking from 9:00pm to 9:00am<br>– Put ashtrays and lighters is separate parts of the house or outside to make smoking less convenient
**Escape**	➢ Leave the situation that causes the craving if possible<br>➢ Not always so easy if you can't leave the children alone, especially if it is children that cause cravings. Try these strategies instead:<br>– Put a movie on for the children<br>– Take a mental vacation, imagine where you would like to be.<br>– Put on headphones and listen to music – you can see the children without hearing them<br>– Keep a treasure box of things from the Two Dollar Shop – if you need a break, a new toy could provide the distraction you require
**Evade**	➢ If possible, avoid places or situations where others will be smoking (when cravings to smoke subside, it will be easier to return to normal routine)<br>➢ Go places where smoking is not allowed e.g. movies, shopping centre, library<br>➢ Hang out with people who are non-smokers, or if not possible, chew gum and take a water bottle to<br>➢ Visit friends who have a non-smoking home
**Distract**	➢ Think of things to distract the mind from smoking, especially things that cannot be done while smoking:<br>– Play a game on your phone<br>– Wash dishes, do your hair, bath the dog<br>– Hammer nails<br>– Peel an orange<br>– Chew gum, seeds or something healthy<br>– Drink water, herbal tea or low carbonated/caffeinated drinks
**Substitute**	➢ When craving, substitute something that keeps hands & mouth busy<br>– Use short acting NRT<br>– Chew NRT gum or regular gum<br>– Chew gum, seeds or something healthy<br>– Brush your teeth

4 PHASE 3 - SUPPORTING SMOKING CESSATION & RELAPSE PREVENTION

4.1 OVERALL AIMS:

- Providing tools and techniques to support changes to smoking behaviour

4.2 GOALS:

- Identify high risk situations
- Strategies to deal with stress
- Dealing with smoking lapse and relapse
- Lifestyle changes to increase long term success
- Reward success

4.2.1 Identify high-risk situations & solutions

Looking forward, looking back

Look back at previous lapses to Identify potential future high-risk situations

Looking Back
➢ Ask participant to think about what they have learnt about themselves in previous attempts to stop smoking (or other substances)<br>➢ Get them to identify the situations or events that occurred just prior to the lapse<br>➢ Identify any themes that emerge e.g. socialising with alcohol, with smoking friends<br>➢ Provide praise for good insight
**Looking Forward**
➢ Ask participant to recognise as many future high-risk situations as they can, based on these past experiences.<br>➢ Are there any new situations that are potential high-risk?<br>➢ Get participant to problem-solve possible solutions to apply to each of these situations.

Linking emotions to solutions

It can be helpful to identify and relate emotions or feelings to high-risk situations before determining strategies to cope. These can then be applied at other times when similar emotions arises. The following are examples only

Situation	Emotion / Feeling	Cigarette-free management
➢ Mixing with friends / relatives / other who smoke cigarettes	➢ Left-out, isolated, alone	➢ Pre-prepare strategy before seeing them<br>➢ Use NRT

		➢ Try mixing with others who don't smoke especially at the start
➢ After eating a meal	➢ Incomplete, nostalgic,	➢ Have a small, relatively healthy treat<br>➢ Use NRT

4.2.2 Strategies to deal with stress

Relationship between stress and nicotine levels

Smokers will often cite stress relief as one of the main triggers for smoking and use cigarettes as a coping mechanism to help calm nerves. In fact, in smokers, feelings of stress are often related to the depletion of nicotine in the blood stream (34). Nicotine levels in the blood decline by half approx. every two hours, creating withdrawal symptoms (e.g. irritability, lack of concentration and cravings) that are similar to feelings of stress and anxiety. These are relieved by a cigarette, creating the impression that smoking relieves the stress. This is illustrated, in simplified terms, by the following diagram:

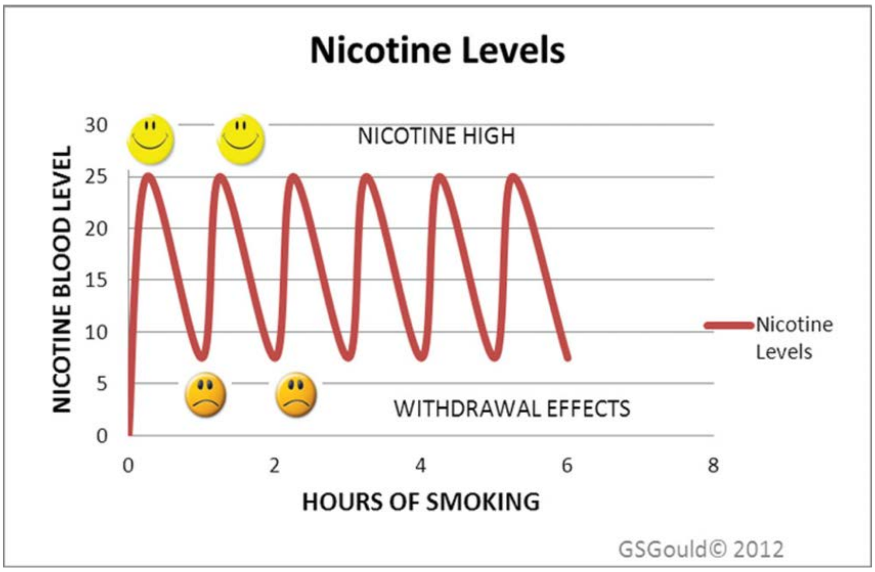

Mindfulness strategies

Recent years have seen an increase in the use of alternative treatments as an aid to smoking cessation including mindfulness techniques. These aim to reduce stress and withdrawal symptoms and can be used as a distraction for cravings. Evidence shows good potential for these as an effective smoking treatment and relapse prevention (35). Following are examples of breathing and visualisation techniques that can be used mindfully:

**Breathing technique**	
1	Find a comfortable quiet place to sit
2	Loosen any tight clothing
3	Close eyes and slow down breathing

4	Take slow, regular, deep breaths
5	Inhale through the nose to a long count of 5, hold for a count of 2, then exhale to a long count of 5
6	Focus on the sounds of your breathing and the feeling of your breath expanding your lungs and diaphragm
7	Continue for about 10 – 15 cycles

**Creative Visualisation**	
1	Find a comfortable quiet place to sit
2	Close eyes and slow down breathing
3	Imagine a picture, place or situation that makes you feel safe, relaxed and happy
4	Breathe in and out slowly and gently through the nose
5	Try and sense the smells, tastes and sounds of the target of your thoughts
6	Feel your body relax
7	Continue for at least 10 minutes

Surfing the Urge: Urges to smoke can be compared to waves in the ocean. Waves start small, get bigger until they peak, then subside and eventually disappear. Learning to ride the urge wave is better than giving into it. The trick is learning to experience the urge mindfully, rather than fighting or suppressing it (36). Urges will decrease in frequency and intensity as time passes.

**Urge Surfing**	
1	Notice the urge. It may be a thought, a craving or a feeling.
2	Accept the feeling, knowing that it is a symptom of withdrawal and that it will subside
3	Sit and breathe through the urge, observing it without responding
4	Focus attention on those areas of the body most effected by the urge e.g. a knot in the stomach, tightness, itchiness.
5	After a short while, the craving will weaken and then completely pass by.

Self-compassion

Self-compassion is the practice of extending compassion and understanding to oneself in times of suffering or when feeling inadequate. It has the ability to lower levels of emotional distress and enhance health and

wellbeing (37). Its ability to increase self-regulation has been shown to reduce levels of smoking in self-critical individuals (38)

Calming Self-talk	
➢	Remind yourself that making changes to tobacco use is difficult and it is normal to feel stressed. These feelings are part of nicotine withdrawal.
➢	Practice self-compassion and saying helpful things to yourself e.g. think about how a friend or family member might support or encourage you in a similar situation and then talk to yourself as they would
➢	Normalise what is happening to you. Almost everyone has similar difficulties when trying to stop smoking, you are not different, weak or stupid.
➢	Ignore or challenge the inner voice that criticises wrongdoings or bad decisions
➢	Practice being kind to yourself with kind affirmations or rewards for achievements, or reaching a set goal

4.2.3 Lapse and relapse in high-risk situations

Pathway of relapse[4]

Highlighting the possible outcomes of a lapse to tobacco and its potential to lead to relapse is useful. The following is a simplified, cognitive-behavioural model that illustrates this process in an easily understandable way. It highlights the different ways of coping after having a cigarette or two, and how this effects a persons self-efficiacy. Self-efficiacy is the belief that an individual has in their ability to succeed in a situation or at a given task, and can influence the liklihood of continuing to smoke. The recognition of a lapse as a slip that can be forgiven and reversed is more likely to lead to a return to no-smoking.

[4] Adapted from Marlatt G and Gordon J. Relapse prevention: maintenance strategies in the treatment of addictive behaviours. New York: Guildford; 1985.

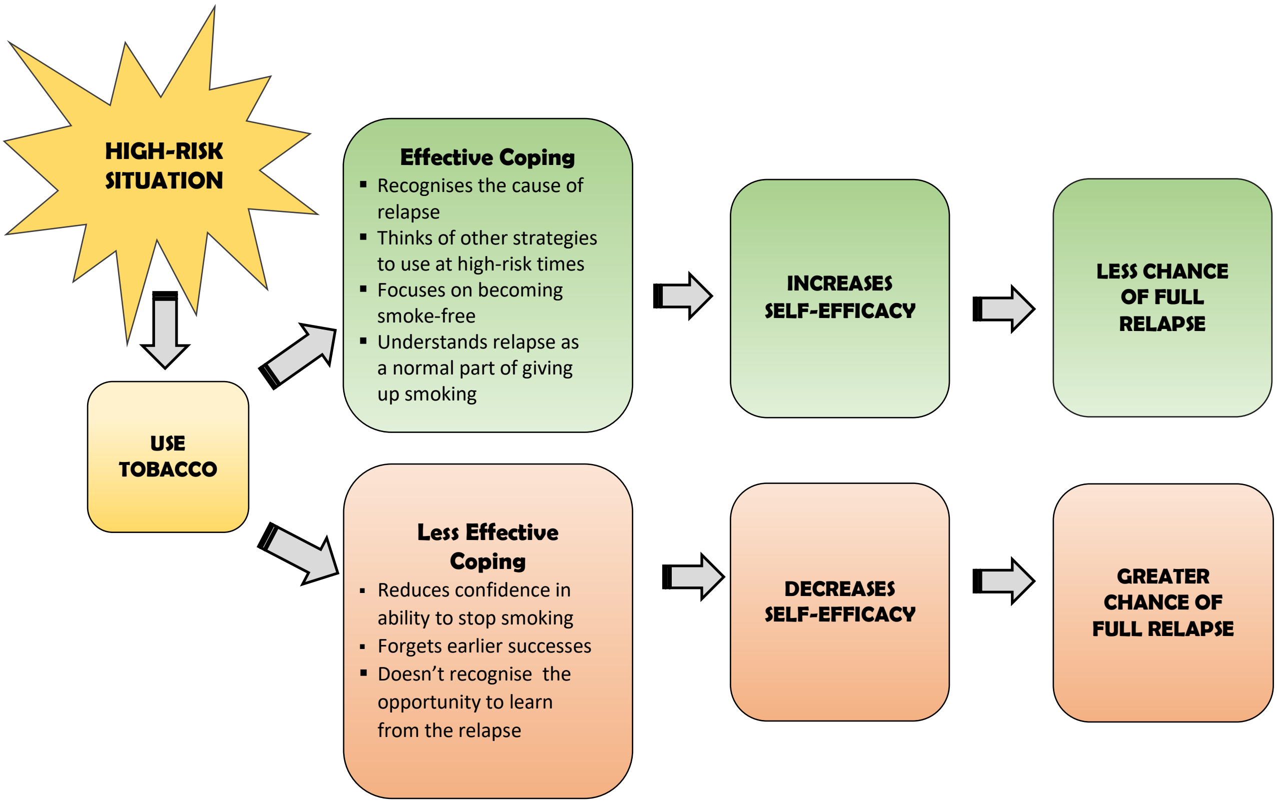

Dealing with cravings

It is common to have strong or constant cravings for cigarettes when making changes to smoking behaviours, especially in the early days. Cravings will pass in 5-10 minutes although strategies exist to cope with them but the important thing is to plan ahead. Identify the situations or feelings that are likely to cause a craving (i.e. triggers), then plan how to deal with them.

**Coping with Cravings**
**Things to Do**
➢ The following list of physical things to do might be helpful:<br>- Use short-acting NRT to combat craving<br>- Switch tasks from the one being done as the craving hit<br>- Talk to a supportive friend or phone a Quitline (Refer to 4.2.4 – *Ongoing Supports*)<br>- Go for a quick walk or take the children to the park<br>- Take deep breaths<br>- Have a drink of water<br>- Chew gum, mints, fennel or anise seeds<br>- Clean your teeth<br>- Do relaxation exercises<br>- Take a shower<br>- Clean the house<br>- Colour in (either alone or with children)<br>- Do something with a non-smoker
**Things to Think**
➢ When it is too difficult to switch tasks, the following list of things to think can help:

Coping with Cravings
- Consider the loss of hard-earned financial incentives from the study<br>- Try mindfulness techniques (Refer to 4.2.2. - *Relaxation, Urge surfing, Visualisation, Calming self-talk*)<br>- Remember a new mantra 'I am a non-smoker'<br>- Attempt to figure out what bought on the craving for a cigarette<br>- Remember that the craving will be over soon<br>- Consider the positive changes that have already made and acknowledge them<br>- Think of protecting children and other house-hold members from second-hand smoke<br>- Think positive thoughts and remember the benefits of not smoking<br>- Don't think of a cigarette as a reward and think of other ways to reward oneself.

Problem solving techniques

Increasing problem solving skills is a cognitive behavioural approach that teaches participants a step-by-step to solving problems associated with lifestyle changes, with evidence showing it to be an effective method for many clinical populations and problems. If has been shown to maintain relapse prevention in smoking cessation studies when compared to other behavioural and control groups (39). It can assist with devising ways to resist the temptation to smoke or help to manage other difficulties that may arise after smoking cessation e.g. coping with the loneliness that comes from avoiding social circles with smokers.

Problem Solving Strategy	
➢ Problem solving gives the opportunity to think about the difficulty, possible alternatives to solve the problem and the best solution. Ask the following questions when faced with a situation with multiple solutions:	
**1**	**What is the problem?**<br>– *Define the problem clearly:*<br>– *E.g. household members are smoking inside and making it difficult to stop smoking*
**2**	**What is my goal?**<br>– *Determine the best possible outcome:*<br>– *E.g. to be smoke free and live happily with other household members*
**3**	**What are my options or alternatives?**<br>– *Think of as many options as possible:*<br>– *E.g. use more NRT to control cravings when others are smoking*<br>– *Use other strategies, such as DEEDS (phase 2) to resist smoking while others are*<br>– *Move out and find a different place to live*<br>– *Suggest that the house become smoke-free inside and set up a smoking area outside*<br>– *Make a designated smoke free area within the house e.g. the kitchen, bedroom etc.*<br>– *Spend more time away from home, with people who don't smoke, at least until smoking urges are under control*
**4**	**Weigh up these options**<br>– *Think about each alternative, weighing the good against the bad for each:*<br>– *e.g. NRT is relatively cheap and easy to use but tastes pretty bad; a smoke free home would be ideal, but housemates might not like to smoke outside; moving out would be expensive but could choose a smoke free home etc.*
**5**	**Select the most suitable option and DO IT!**

Conflict resolution

Withdrawal from cigarettes can be associated with irritability and anger. These feelings are likely to be strongest during the early stages of cessation. During this time, conflict might occur with partners, other household members or people from social networks. Having some pre-thought-out strategies in place to avoid conflict, or, if it does occur, resolve it, will reduce stress levels and minimise the chance of relapse.

**Tips to Avoid Conflict**
➢ Use NRT to minimise withdrawal symptoms including irritability, mood swings etc.
➢ Prepare partner and/or those around you about the potential for intense emotions due to nicotine withdrawal and ask for their understanding.
➢ Avoid situations that have the potential to induce conflict
➢ Practice and use relaxation and grounding techniques
➢ Use distraction techniques (refer to 3.2.4 - *DEEDS strategy*)
**Tips for Resolving Conflict**
➢ Compromise – be willing to bend and meet the other party half way
➢ Use humour – used respectfully, humour can diffuse heated situations. Focusing on oneself can be effective
➢ Apologise – people tend to lose the desire to remain in conflict once an apology has been issued.
➢ Share the responsibility – for both the conflict and its solution.
➢ Pick your battles – minor conflict is often not worth the effort required to resolve it.
➢ Get help – if conflicts cannot be resolved, mediation or advice from a neutral third party can be beneficial

Grounding technique:

Grounding is a mindfulness technique useful when feeling overwhelmed, distracted, anxious or angry. It is helpful to re-orient to the present and regain mental focus after being in an intense emotional state such as those that may be bought up in the early stages of nicotine withdrawal.

**Grounding technique**
Find the following:
➢ **5** things you can **SEE**
➢ **4** things you can **HEAR**
➢ **3** things you can **TOUCH**
➢ **2** things you can **SMELL**
➢ **1** deep **BREATH**

4.2.4 Ongoing support:

Remind participant of existing social supports that could have been discussed in earlier sessions; these are ones available within her social environment. External supports also exist and are useful if chosen support networks are unavailable or not helpful. The following table lists some of the more well-known public supports available.

External quit smoking support providers

**Quitlines**
➢ **NSW Quitline** - 13 7848 (13 QUIT)<br>➢ **NSW Aboriginal Quitline** - 13 7848. ask to speak to the NSW Aboriginal Quitline<br>➢ **Multicultural Quitline**– see https://www.icanquit.com.au for times available<br>– Arabic - 1300 7848 03<br>– Vietnamese - 1300 7848 65<br>– Chinese - 1300 7848 36
**Smoking apps**
➢ **My Quit Buddy** – tips & distractions, tracking, progress graphs, facts,<br>➢ **Quit for you - Quit for two** – pregnancy focused, inspirational, fun exercises & games, personalised<br>➢ **Quittr** – includes fun games, personalised statistics, behavioural support<br>➢ **Stop Smoking - Mindfulness Meditation** – provides meditation/relaxation techniques

4.2.5 Promote long-term success:

Strategies for a healthy lifestyle

Smoking cessation and pregnancy provide the perfect opportunity to start focusing on good health. These improvements will help to maintain the motivation to remain smoke-free.

**A Healthy Lifestyle**
➢ Weight gain can be a concern for many smokers, particularly women.<br>➢ The average weight gain after cessation is approx. 4-5 kg, although not everyone who stops smoking will gain weight (40).<br>➢ The health benefits of not smoking outweigh the risk of a few extra kilo's, so quitting is a good time to think about making changes to diet, exercise and other aspects of health.<br>➢ Once you see improvements in one area, it is easier to make changes in others. The benefits are far reaching and can include:<br>– Healthier pregnancy<br>– Improvements to health of other children<br>– Greater coping and improved mental health<br>– Better sleep<br>– Improved relationships<br>– Better sex<br>– More confidence<br>– Increases long-term happiness
**Diet**

A Healthy Lifestyle
➢ A healthy diet helps prevent weight gain<br>➢ Hunger is a withdrawal symptom, be prepared:<br>– snack on right foods e.g. fruit, nuts, carrots, yoghurt, boiled eggs, banana smoothie<br>– if possible, eat smaller meals more often<br>➢ Eat a good breakfast:<br>– morning cigarette cravings aren't as strong with a full stomach<br>– it improves mental alertness, concentration and mood, all things that are affected when withdrawing from cigarettes<br>➢ Food preparation is a distraction from smoking
**Exercise**
➢ Helps prevent weight gain by decreasing appetite<br>➢ Excellent for releasing brain endorphins that improve stress, anxiety and mood<br>➢ Great distraction from thoughts of smoking<br>➢ Eases withdrawal symptoms & cravings to smoke during and after exercising
**Relaxation**
➢ Great for relieving everyday stress particularly that associated with not smoking<br>➢ Great distraction from thoughts of smoking<br>➢ Try the following to relax:<br>– Deep breathing relaxation technique or creative visualisation listed in this manual<br>– Listening to music<br>– Squeezing a stress ball<br>– Practicing mindfulness / meditation<br>– Colouring in<br>– Walking

Sleep Improvement

Sleep disturbances are a common side-effect of smoking cessation, whether it is difficulty getting to and staying asleep or feeling drowsy during the day. The use of some cessation aids such as NRT patches has also been associated with sleep disturbances during cessation (41).

Sleep Hygiene
➢ Sleep hygiene is a term used to describe healthy sleeping habits.<br>➢ For the first few weeks of not smoking, it is common to have some trouble sleeping at night as your body goes through nicotine withdrawal.
**Tips for creating good sleep hygiene:**

Sleep Hygiene
➢ Avoid caffeine and any other chemicals that interrupt with sleep<br>➢ Go to bed at the same time each night to create a regular bedtime<br>➢ Create a dark, quiet and cool bedroom environment e.g. use sleep mask, earplugs<br>➢ Only use the bed for sleep & sex. Don't watch TV, eat or read from bed.<br>➢ Don't have sleeps during the day, or, have a short nap before 3:00pm (no more than 20 minutes)<br>➢ Have a warm bath before bed to help create sleepiness<br>➢ Don't watch the time during the night as this helps keep us alert and awake.<br>➢ Get some sunlight during the day to help create a healthy sleep-wake cycle<br>➢ If unable to sleep after 20 minutes, get up and do something relaxing until feeling tired enough to go to sleep<br>➢ If awake during the night & not able to go back to sleep, get up, keep the lights dim and do something non-stimulating such as listening to soft music or reading a telephone book for 20 minutes, then try again.<br>➢ Eat well during the day but avoid a heavy meal before bed. Have a glass of warm milk instead.<br>➢ Exercise during the day (preferably in the morning) as this helps to increase tiredness at night.

Drug interactions with smoking cessation

Levels of medication can vary in the body of someone who stops smoking or changes how much they smoke. This is because cigarette smoking increases the activity of certain enzymes involved with the breakdown of some medications, so a change in smoking alters the body's metabolism of them. The following is a guide[5] to the medications involved. Advice should be sought from a GP.

Note that nicotine replacement therapy does NOT affect medication levels. Changes are caused primarily by the elements of tobacco smoke rather than nicotine itself.

Drug	Effect of smoking cessation	Impact on dosage after cessation	Clinical importance
Benzodiazepines	Possible increased sedation due to loss of CNS stimulation by nicotine.	May need lower dose. May be more sedated if dose remains the same	+
Beta blockers	Serum levels rise and effects enhanced.	May need lower dose.	+
Caffeine & alcohol	Caffeine levels rise<br>Alcohol levels rise	Reduce caffeine and alcohol levels by half within a week	+++
Chlorpromazine	Serum levels rise	May need lower dose	+
Clopidogrel	Effectiveness is significantly reduced when smoker stops smoking	Prasugrel or ticagrelor may be better choices for non-smokers	+++
Clozapine	Serum levels rise significantly	An average 50% dose reduction may be required	+++
Flecainide	Serum levels rise	May need lower dose	+
Fluvoxamine	Serum levels rise	May need lower dose	+
Haloperidol	Serum levels rise	May need lower dose	+
Heparin	Serum levels rise	May need lower dose	+

[5] Adapted from NSW Ministry of Health, Quick guide to drug interactions with smoking cessation (2016).

Drug	Effect of smoking cessation	Impact on dosage after cessation	Clinical importance
Imipramine	Serum levels rise - monitor for side effects	May need lower dose	+
Insulin	Increased subcutaneous absorption<br>due to vasodilation after quitting	May need lower dose	++
Olanzapine	Serum levels rise significantly	An average 30% dose reduction may be required	+++
Theophylline	Serum levels rise	May need lower dose	++
Warfarin	Serum levels increase by 15% on average	May need lower dose. Close monitoring of INR advised.	+++

4.2.6 Reward Success

It is well established that financial rewards are one of the most effective ways to motivate people to stop smoking, especially pregnant smokers (42). The iQuiP study provides rewards in return for smoking reductions and abstinence, but these are time-limited and there is a risk of relapse to smoking when rewards stop in the postpartum period.

Rewards work by being a natural mood-lifter. During cessation, the brain must adapt to not having nicotine to trigger the release of the feel-good chemical dopamine. A reward (financial or otherwise) can improve mood by releasing this neurotransmitter and train the brain that reward is associated with something other than cigarettes.

Encourage participants to think of other ways that they can celebrate their hard work and success while on the study and afterwards. Motivators are different for every person, but the following suggestions can be tailored to the individual.

Rewards for success
➢ Simple, inexpensive, everyday rewards might include having a lie-in, listening to music, going for a walk, reading a book, watching a movie, eating some favourite food, or having a warm, relaxing bath.<br>➢ Calculate the potential amount of savings from not smoking between now and next session. Determine how this money could be used to enhance quality of life.<br>➢ Try putting the equivalent of a packet of cigarettes into a separate account each week. Have a savings goal e.g. a holiday or short escape, a trip to the theme park, buying new clothes after baby is born<br>➢ Eat out or order a nice take-away for a mid-week treat. The food will taste better and the break from cooking is appreciated more with a new baby or one on the way.<br>➢ See a movie, either alone, with a partner, or with children. It should be easier to sit through a movie without wanting a cigarette.<br>➢ Ask someone to look after children (if necessary) and book a massage.

5 REFERENCES

1. Urquhart C, Jasiura F, Poole N, Nathoo T, Greaves L. Liberation! Helping women quit smoking: a brief tobacco-intervention guide. Vancouver BC,: British Columbia Centre of Excellence for Women's Health; 2012.

2. Gould GS, Bittoun R, Clarke MJ. A Pragmatic Guide for Smoking Cessation Counselling and the Initiation of Nicotine Replacement Therapy for Pregnant Aboriginal and Torres Strait Islander Smokers. J Smok Cessat. 2015;10(2):96-105.

3. Greaves L, Poole N, Chizimuzo T, Hemsing N, Qu A, Bialystok L, et al. Expecting to quit: a best-practices review of smoking cessation interventions for pregnant and postpartum girls and women. Vancouver: British Columbia Centre of Excellence for Women's Health; 2011 [cited 20 Jan 2019]. Available from: http://works.bepress.com/chizimuzo_okoli/61/.

4. Stephen Rollnick, William R Miller. Motivational interviewing: Preparing people to change addictive behavior. New York: Guilford Press; 1991.

5. Hemsing N, Greaves L, Poole N. Tobacco cessation interventions for underserved women. Journal of Social Work Practice in the Addictions. 2015;15(3):267-87.

6. Lindson-Hawley N, Thompson TP, Begh R. Motivational interviewing for smoking cessation. Cochrane Database of Systematic Reviews. 2015(3).

7. Pollak KI, Oncken CA, Lipkus IM, Lyna P, Swamy GK, Pletsch PK, et al. Nicotine replacement and behavioral therapy for smoking cessation in pregnancy. Am J Prev Med. 2007;33(4):297-305.

8. Perkins KA, Conklin CA, Levine MD. Cognitive-behavioral therapy for smoking cessation: A practical guidebook to the most effective treatments. New York, NY, US: Routledge/Taylor & Francis Group; 2008. xxii, 258-xxii, p.

9. Diclemente CC. Failure to change or failure to sustain: pregnancy smoking and postpartum abstinence. Addiction. 2016;111(6):992-3.

10. Gamble J, Grant J, Tsourtos G. Missed opportunities: a qualitative exploration of the experiences of smoking cessation interventions among socially disadvantaged pregnant women. Women Birth. 2015;28(1):8-15.

11. Oxford University Press [Internet]. English Oxford Ddictionaries 2018 [20 June 2018]. Available from: https://en.oxforddictionaries.com/definition/stigma.

12. Kelly JF, Saitz R, Wakeman S. Language, substance use disorders, and policy: the need to reach consensus on an "addiction-ary". Alcoholism Treatment Quarterly. 2016;34(1):116-23.

13. C. Duong, N. Perry, A. Brown, S. Ling, A. Drury, J. Byrne, et al. Changes in Assumption of Care and Primary Drug Use in Substance Use in Pregnancy Service . Clin Case Rep Rev [Internet]. 2017.

14. Twyman L, Bonevski B, Paul C, Bryant J. Perceived barriers to smoking cessation in selected vulnerable groups: A systematic review of the qualitative and quantitative literature. BMJ Open. 2014;4(12).

15. Fallin A, Miller A, Ashford K. Smoking Among Pregnant Women in Outpatient Treatment for Opioid Dependence: A Qualitative Inquiry. Nicotine & Tobacco Research [Internet]. 2016; 18(8):[1727-32 pp.]. Available from: http://dx.doi.org/10.1093/ntr/ntw023.

16. Acquavita SP, Talks A, Fiser K. Facilitators and barriers to cigarette smoking while pregnant for women with substance use disorders. Nicotine Tob Res. 2017;19(5):555-61.

17. Benowitz NL. Nicotine Addiction. The New England journal of medicine. 2010;362(24):2295-303.

18. Mendelsohn C, Wodak Am A. Smoking cessation in people with alcohol and other drug problems. Aust Fam Physician. 2016;45:569-73.

19. Prochaska JJ, Delucchi K, Hall SM. A meta-analysis of smoking cessation interventions with individuals in substance abuse treatment or recovery. J Consult Clin Psychol. 2004;72(6):1144-56.

20. Thurgood SL, McNeill A, Clark-Carter D, Brose LS. A systematic review of smoking cessation interventions for adults in substance abuse treatment or recovery. Nicotine Tob Res. 2016;18(5):993-1001.

21. Tsoh JY, Chi FW, Mertens JR, Weisner CM. Stopping smoking during first year of substance use treatment predicted 9-year alcohol and drug treatment outcomes. Drug Alcohol Depend. 2011;114(2-3):110-8.

22. Weinberger AH, Platt J, Jiang B, Goodwin RD. Cigarette smoking and risk of alcohol use relapse among adults in recovery from alcohol use disorders. Alcohol Clin Exp Res. 2015;39(10):1989-96.

23. Aschbrenner KA, Naslund JA, Gill L, Hughes T, O'Malley AJ, Bartels SJ, et al. Qualitative analysis of social network influences on quitting smoking among individuals with serious mental illness. Journal of Mental Health. 2017:1-7.

24. Bernstein IM, Mongeon JA, Badger GJ, Solomon L, Heil SH, Higgins ST. Maternal smoking and its association with birth weight. Obstet Gynecol. 2005;106(5 (Pt 1)):986-91.

25. Lindson-Hawley N, Aveyard P, Hughes JR. Reduction versus abrupt cessation in smokers who want to quit. Cochrane Database Syst Rev. 2012(11).

26. Zawertailo L, Ragusila A, Voci S, Ivanova A, Baliunas D, Selby P. Target quit date timing as a predictor of smoking cessation outcomes. Psychol Addict Behav. 2017;31(6):655-63.

27. Beard E, Bruguera C, McNeill A, Brown J, West R. Association of amount and duration of NRT use in smokers with cigarette consumption and motivation to stop smoking: A national survey of smokers in England. Addict Behav. 2015;40:33-8.

28. Bar-Zeev Y, Lim LL, Bonevski B, Gruppetta M, Gould GS. Nicotine replacement therapy for smoking cessation in pregnancy. Med J Aust. 2018;208(1):46-51.

29. Coleman T, Chamberlain C, Davey M, Cooper SE, Leonardi-Bee J. Pharmacological interventions for promoting smoking cessation during pregnancy. Cochrane Database of Systematic Reviews. 2015(12).

30. Wickström R. Effects of nicotine during pregnancy: human and experimental evidence. Curr Neuropharmacol. 2007;5(3):213-22.

31. Benowitz NL, Dempsey DA. Pharmacotherapy for smoking cessation during pregnancy. Nicotine Tob Res. 2004;6(Suppl_2):S189-S202.

32. Bowker K, Lewis S, Coleman T, Cooper S. Changes in the rate of nicotine metabolism across pregnancy: a longitudinal study. Addiction (Abingdon, England). 2015;110(11):1827-32.

33. Hsia SL, Myers MG, Chen TC. Combination nicotine replacement therapy: strategies for initiation and tapering. Prev Med. 2017;97:45-9.

34. Parrott AC. Does cigarette smoking cause stress? Am Psychol. 1999;54(10):817-20.

35. Oikonomou MT, Arvanitis M, Sokolove RL. Mindfulness training for smoking cessation: A meta-analysis of randomized-controlled trials. J Health Psychol. 2017;22(14):1841-50.

36. Bowen S, Chawla N, Collins SE, Witkiewitz K, Hsu S, Grow J, et al. Mindfulness-Based Relapse Prevention for Substance Use Disorders: A Pilot Efficacy Trial. Substance Abuse. 2009;30(4):295-305.

37. Neff K. Self-Compassion: An Alternative Conceptualization of a Healthy Attitude Toward Oneself. Self and Identity. 2003;2(2):85-101.

38. Kelly AC, Zuroff DC, Foa CL, Gilbert P. Who benefits from training in self-compassionate self-regulation? A study of smoking reduction. J Soc Clin Psychol. 2010;29(7):727-55.

39. Karol RL, Richards CS. Making treatment effects last: an investigation of maintenance strategies for smoking reduction. Annual Convention of the Association for Advancement of Behavior Therapy; Chicago1978.

40. Aubin H-J, Farley A, Lycett D, Lahmek P, Aveyard P. Weight gain in smokers after quitting cigarettes: meta-analysis. BMJ : British Medical Journal. 2012;345.

41. Colrain IM, Trinder J Fau - Swan GE, Swan GE. The impact of smoking cessation on objective and subjective markers of sleep: review, synthesis, and recommendations. Nicotine Tob Res. 2004;6(6):913-25.

42. Chamberlain C, O'Mara-Eves A, Porter J, Coleman T, Perlen SM, Thomas J, et al. Psychosocial interventions for supporting women to stop smoking in pregnancy. Cochrane Database Syst Rev. 2017(2).

6 APPENDIX

6.1 SESSION GUIDE - (*DOUBLE CLICK TO OPEN PDF VERSION*)

Participant ID: ______________________________ **Participant Site:** ______________________________

Date: __________ / __________ / __________ **Session Number:** ______________________________

Start time: ______________________________ **am / pm**

Incentives to Quit tobacco in Pregnancy - Counselling for Smoking Cessation

This form is to be completed for each counselling session and used in conjunction with the Counsellors Guide.

Check off what has been discussed in the current session:

Engaging women in change	✓	Guiding cessation	✓	Supporting cessation & relapse prevention	✓
2.2.1 Establish rapport	☐	3.2.1 Increase motivation to change		4.2.1 Identify high-risk situations	
2.2.2 Housekeeping	☐	- Decisional balance	☐	- Looking forward, looking back	☐
2.2.3 Relationship with tobacco		- Reframing techniques	☐	- Link emotions to solutions	☐
- Smoking history	☐	- Motivational question guide	☐	4.2.2 Strategies to deal with stress	
- Barriers to addressing smoking	☐	3.2.2 Identify Social Supports	☐	- Stress and nicotine withdrawal	☐
2.2.4 Stigma reduction		3.2.3 Benefits of Smoking cessation		- Mindfulness strategies	☐
- Personal strengths	☐	- Baby health	☐	- Self-compassion	☐
- Positive health strategies	☐	- Maternal health	☐	4.2.3 Lapse and relapse Prevention	
2.2.5 Tobacco addiction education		3.2.4 Preparing to stop smoking		- Pathway of relapse	☐
- Understanding tobacco dependence	☐	- Reduce tobacco intake	☐	- Dealing with cravings	☐
- Effect of nicotine on the brain	☐	- Cigarette fading	☐	- Problem solving techniques	☐
- Nicotine addiction cycles	☐	- Using NRT	☐	- Conflict resolution	☐
- Harm causing agents in cigarettes	☐	- Resisting urges to smoke	☐	- Grounding technique	☐
2.2.6 Understanding dual dependence	☐			4.2.4 Ongoing support	
				- External smoking support providers	☐
				4.2.5 Promote long-term success	
				- Strategies for a healthy lifestyle	☐
				- Sleep Improvement	☐
				- Drug interactions & cessation	☐
				4.2.6 Reward Success	☐

Where is participant at with their smoking today? Praise successes and offer encouragement as required.

____

____

____

Cravings and urges: Assess cravings to smoke tobacco and link to workbook as required (e.g. DEEDS strategy)

____

____

____

Contingency management: Discuss how Incentives are working to increase motivation to or maintain smoking cessation

____

____

____

NRT adherence: Discuss and offer advice on NRT products being used and how

____

____

____

NRT side effects: Talk over any concerns or problems with NRT and suggest solutions

____

____

____

Goals setting for smoking reduction and cessation: Discuss reduction success and attempts to reduce number of cigarettes to zero

____

____

____

Target date: Discuss and (if possible) set a date for reduction of cigarettes to zero

____

____

Session conclusion

Motivational assessment

How important is it for you to make/maintain changes to your smoking right now?

0	1	2	3	4	5	6	7	8	9	10
Not at all important									Very important	

How confident are you that you can make/maintain this change?

0	1	2	3	4	5	6	7	8	9	10
Not at all confident									Very confident	

Summarise session and together design home tasks and set an agenda for the next session

____

____

____

____

Time and date for next appointment____

Finish Time: ________________ **am / pm** **Counsellor Initials:** ________________________
