# Supplementary material for: Tobacco treatment incorporating contingency management, nicotine replacement therapy, and behavioral counseling for pregnant women who use substances: a feasibility trial
Source: Front Psychiatry. 2023 Aug 16;14:1207955. doi: 10.3389/fpsyt.2023.1207955 (PMC10467262; doi:10.3389/fpsyt.2023.1207955)
Supplement: Supplementary Data Sheet 5 — Smoking cessation - A guide for participants. [file Data_Sheet_5.PDF]

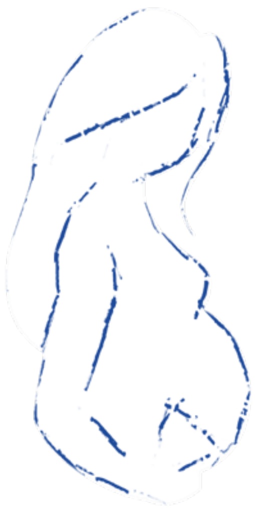

Incentives to Quit tobacco in Pregnancy

(iQuiP)

SMOKING CESSATION SUPPORT

A guide for Participants

Contents

Phase 1 - Engaging in the change process ... 3
Relationship with Tobacco ... 3
Smoking History ... 3
Tobacco addiction education ... 4
Understanding the physical and psychological dependence to tobacco ... 4
Effect of nicotine on the brain ... 5
Nicotine addiction cycles ... 5
Harm causing agents in cigarettes ... 6
Quitting two substances at once ... 7
Phase 2 - Guiding Cessation ... 8
Pros and Cons of Smoking Tobacco ... 8
Reframing ... 9
Identify social supports ... 10
Benefits of smoking cessation ... 11
Preparing to stop smoking ... 12
Resisting the smoking urge ... 13
Phase 3 - Supporting smoking cessation & relapse prevention ... 14
Identify high-risk situations & solutions ... 14
Looking back, looking forward ... 14
Linking emotions to strategies ... 14
Strategies to deal with stress ... 15
Relationship between stress and nicotine levels ... 15
Mindfulness strategies ... 15
Self-compassion ... 16
Lapse and relapse in high risk situations ... 17
Pathway of relapse ... 17
Dealing with cravings ... 17
Problem solving techniques ... 18
Conflict resolution ... 19
Grounding technique: ... 19
Ongoing support ... 20
External quit smoking support providers ... 20
Promote long-term success: ... 20
Sleep Improvement ... 20
Strategies for a healthy lifestyle ... 21
Reward Success ... 22
Notes ... 23

Phase 1 - Engaging in the Change Process

Relationship with tobacco

Smoking history

Why do I smoke? ____

____

____

____

What are the good things about smoking? ____

____

____

____

What are the negatives aspects of smoking? ____

____

____

____

What are my triggers for smoking? *Think about coffee, alcohol, social situations* _______________________

____

____

____

What is preventing me from stopping cigarettes? ____

____

____

____

Tobacco addiction education

Understanding the physical and psychological dependence to tobacco

Addiction to Tobacco Smoking

Physical Dependence

- This is the addiction to nicotine.
- The body needs nicotine to function normally
- Without nicotine, the body goes to nicotine withdrawal
- Withdrawal symptoms occur. These can include
  - Cravings or urges to smoke
  - Irritability, anger, anxiety, sadness, depression
  - Restlessness or difficulty concentrating
  - Sleeping difficulties or disturbances
    - Increase in hunger

Providing the body with nicotine can reduce these symptoms

Psychological Dependence

This is the habit of smoking, triggered by the cues that have been repeatedly linked to smoking

These cues can be:

- *Situational* e.g. smoking with coffee or alcohol
- *Emotional* e.g. smoking when feeling stressed
- *Visual* e.g. seeing someone else smoking
- *Physical* e.g. smelling cigarette smoke or having nothing to do with your hands

Cues create urges to smoke

Identifying the individual cues that trigger smoking is the first step in breaking the tobacco

Nicotine Replacement Therapy

Behavioural Treatments

Treatment for Tobacco Smoking

Effect of nicotine on the brain

Nicotine is the active ingredient in cigarettes that causes addiction. It improves memory and concentration, increases heart rate and reduces appetite.

It enters the brain through the brain stem and activates the area known as the reward or pleasure centre. Here it releases large amounts of the 'feel good' hormone dopamine, a chemical that helps to reinforce behaviours that are pleasurable.

Generally, nicotine levels peak around 10 minutes after inhalation them but disappear quickly, creating the continual need to smoking throughout the day.

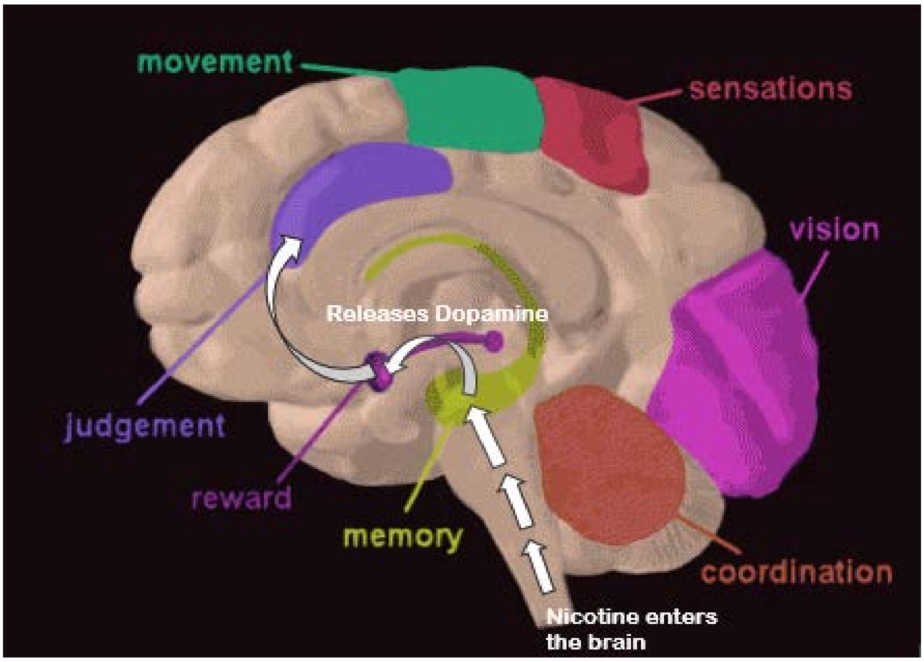

Nicotine addiction cycles

This figure shows the typical withdrawal from nicotine that a smoker goes through every day. The up-points show nicotine levels after a cigarette and each low point shows how quickly nicotine levels drop (this takes around 30 to 40 minutes).

The points that reach outside the top nicotine tolerance level show pleasure. Those that reach below the lower tolerance level show withdrawal. The first cigarette of the day has the most pleasurable effect. The area between the two horizontal lines represents the area that a smoker feels comfortable without experiencing pleasure or withdrawal.

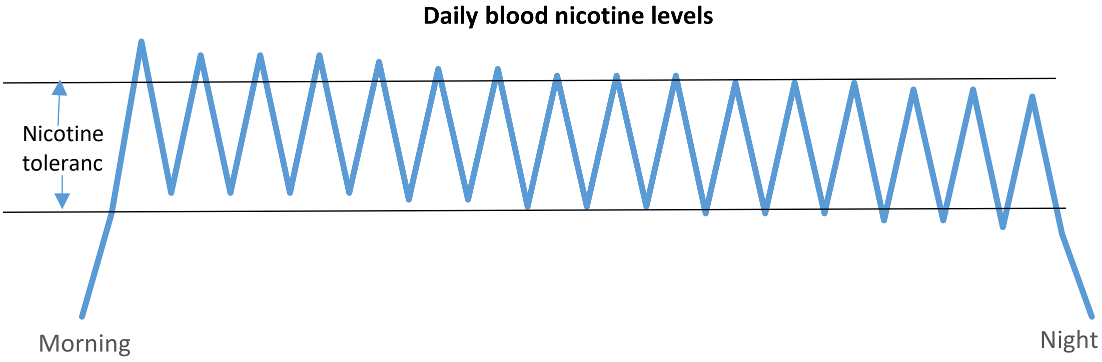

Harm causing agents in cigarettes

Cigarettes contain over 7000 chemicals that are ingested into the blood stream with every cigarette smoked

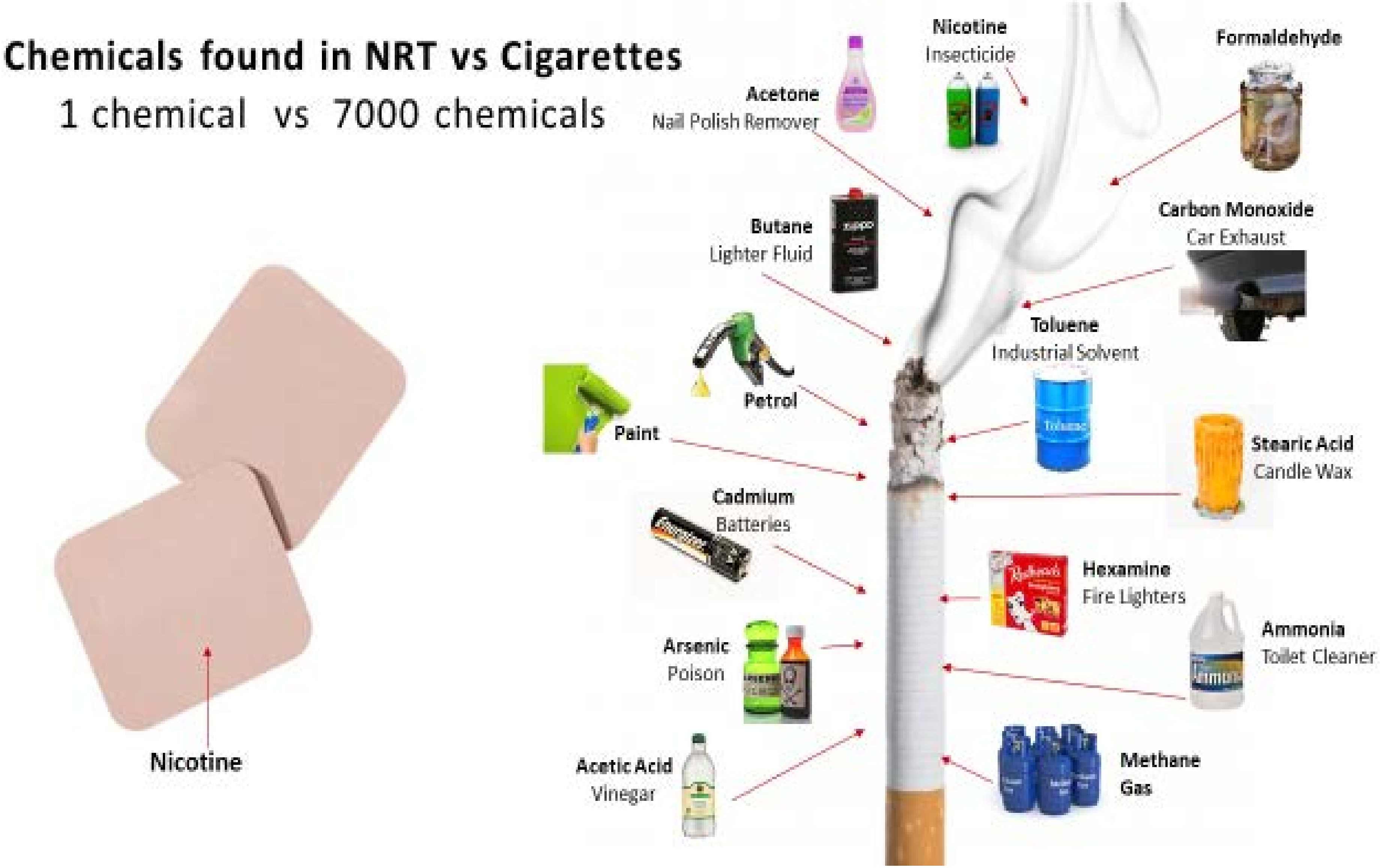

Quitting two substances at once

It is not uncommon to have two addictions at the one time; two out of three people in Australia with an alcohol or other drug disorder also smoke. It was not commonly encouraged to give up two substances at once (e.g. cigarettes and pot) but research is showing that giving up one can improve the ability to give up the other The following table shows some of the myths and evidence for stopping both at once.

Smoking while using other Drugs	
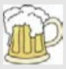 **Alcohol**	➢ Nicotine and alcohol increase the enjoyment of each other<br>➢ Drinking alcohol increases the urge to smoke because alcohol is a common trigger<br>➢ Smoking can also increase the urge to drink alcohol<br>➢ Smoking reduces the effects of alcohol e.g. light-headedness, tiredness, slurred speech, poor coordination etc.
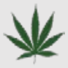 **Cannabis**	➢ Two out of three people who use cannabis, spin it with tobacco<br>➢ This leads to higher levels of nicotine dependence<br>➢ Quitting both together is recommended as continuing to spin tobacco in cannabis can make quitting tobacco harder<br>➢ Cannabis and tobacco have similar withdrawal symptoms and the combined symptoms may be more severe<br>➢ The same strategies used to stop smoking can also help reduce or stop cannabis
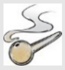 **Stimulants**	➢ More than 80% of people who use stimulants (speed, ice, MDMA, cocaine) smoke tobacco<br>➢ Cocaine use increases cigarette smoking and nicotine use increases cocaine use<br>➢ Stopping cigarettes helps to reduced cravings for stimulants<br>➢ Quitting smoking while having stimulant treatment can make smoking cessation easier and more successful
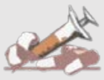 **Opiates**	➢ People who use opiates including heroin, methadone and buprenorphine have the highest rates of smoking.<br>➢ People who use methadone generally smoke more heavily in the four hours after each dose.<br>➢ Nicotine can weaken some side-effects of methadone such as sleepiness and loss of concentration

Stopping two substances together	
**Myths**	➢ Smokers who use other substances are not able to quit, so helping them to stop smoking is a waste of time and resources<br>➢ Smokers who use other substances don't want to quit smoking<br>➢ Smoking is less dangerous or less important than other addictive substances<br>➢ Stopping two substances at once is too difficult
**Evidence**	➢ People seeking treatment for substance use want to stop smoking tobacco at similar rates to the general population of smokers<br>➢ People treated for substance use and smoking together are 4 times more likely to be not using alcohol and other drugs in the long-term than those who don't receive smoking treatment<br>➢ Those who quit smoking during their first year of substance use treatment are more likely to be not using alcohol &/or other drugs after 9 years than those who didn't<br>➢ Continued smoking can cause people to restart alcohol and/or other drugs after they had stopped using them

Phase 2 - Guiding Cessation

Pros and cons of smoking tobacco

	Benefits / Pros of smoking	Costs / Cons of smoking
**No Change in smoking**	______<br>______<br>______<br>______<br>______<br>______	______<br>______<br>______<br>______<br>______<br>______
**Change in smoking**	______<br>______<br>______<br>______<br>______<br>______	______<br>______<br>______<br>______<br>______<br>______

Reframing

Reframing is a way of changing think about some of the more common justifications of smoking

Common thoughts	Responses
Smoking is the least of my problems	Often tackling one problem can benefit other areas or problems. For example, those who stop smoking are more likely to stay off other substances. Also, staying off cigarettes can save money and help relieve financial stress
It is too difficult to quit two substances at once	Yes it is difficult, however people who have stopped report that it is easier to use no substances than stop one at a time. This is because smoking is often linked to the other substance use e.g. drinking alcohol and smoking, spinning cannabis with tobacco. Research has found that people who stop smoking are more likely to stay off other substance. Continuing to smoke increases the chances or relapsing to other substances
I need cigarettes to help me cope or relieve my stress	People who smoke think that smoking relieves anxiety and stress yet stopping smoking can help people cope better. Part of the stress felt is the body going into nicotine withdrawal between cigarettes. Most people will find that stress levels improve and they can cope better within 6-weeks of giving up. Working on mindful stress reduction techniques such as breathing and visualisation can be very helpful.
Smoking is the lesser of two evils	The problem about smoking is that the physical damage is not experienced until much later on. The damage experienced by alcohol and other substances can be felt much sooner e.g. overdose, criminal conviction
I can't give up everything…smoking is my only remaining vice	Some people believe that smoking gives pleasure. Pleasure is more likely to be from the situations where they smoke e.g. with friends, or from taking time out to have a cigarette. Everyone deserves activities that provide pleasure, so think about other ways you can experience this.
I don't want to use NRT as I will still be addicted to nicotine.	It is the smoke from burning tobacco and the chemicals within cigarettes that cause significant health damage, not nicotine. It is rare for people to become addicted to nicotine replacement products, but for those that do, it is less expensive and much less harmful than tobacco smoking.
Smoking is just a habit, a way of life	Smoking is both a physical addiction and a psychological dependence. Breaking the habit of smoking is very important, however, it is also important to treat the addiction and symptoms of withdrawal.
No one has bothered about my smoking before	It was not uncommon for tobacco smoking to be viewed as less important when treating substance use or mental health. It was thought that stopping tobacco use would cause people to return to other substances. We now know that treating smoking can improve substance use treatment.
It's too late, the damage from smoking & using drugs is already done	It's never too late to stop smoking and the benefits to health are immediate. Most people who stop smoking will feel better physically and mentally in the long run.
It's not worth the hassle to stop smoking	It does seem like hard work but stopping makes such a positive difference to your health, and assists during pregnancy, that any attempt to reduce smoking is worth it.
I don't care if it kills me, you have to die of something	This may be true, but death from smoking is preventable and giving up will give you more years of living. Everyone deserves the financial and health benefits of not smoking,

Common thoughts	Responses
I'm so bored now and I always smoke when I'm bored	It is common to feel bored when cutting down or stopping smoking for good. It is a good idea to identify things that you enjoy that can make you feel less bored. (Refer to 3.2.4 DEEDS Strategy)
It's all too much and too hard	Yes it is difficult because you are making changes to smoking, and nicotine is an addictive and powerful drug. Also remember that withdrawal symptoms and cravings get a little easier to handle every day, until they eventually stop for good. When feeling that it is all too hard, consider using NRT, speaking to your social supports. It might also help to focus on the financial incentives and health benefits gained from not smoking.
It's not the same (without cigarettes)	There is adjustment associated with stopping any habit. It might feel like there is more time in the day, or you have a sense of loneliness. It is common to feel 'different' without the cigarettes that have previously taken up a large part of life.
Smoking is my best friend	Smoking can feel like a friend, giving support when you need it. It is normal to have feelings of loss when you stop, with the void created by not smoking often interpreted as loneliness. It is worth wondering whether a friend would cause as much harm, and cost as much money, as cigarettes do.
I've tried to quit before and I failed	It is common to have many attempts at stopping before being successful. Each of these attempts is a valuable lesson about relapse triggers, and knowing these can make the next attempt more successful. Combining strategies, like using NRT with counselling support and financial incentives can help.

Identify social supports

Spending time socialising with friends or working with groups of people who smoke makes it difficult to stop smoking and increases the risk of relapse, especially in the early days of giving-up. Spending less time with these high-risk groups and more time with supportive people who don't smoke, at least for a while, can lead to greater success.

Are there friends and/or family members who are aware of the participant wanting to stop smoking that could provide support during the quitting process?

____

____

____

____

Do you have other recreational interests or hobbies that could help distract and keep you occupied during the quitting process?

____

____

____

____

Benefits of smoking cessation

Smoking and Pregnancy

- Optimal fetal development requires a healthy placenta joining a baby to its mother in the womb.
- The placenta is vital for supplying all the oxygen, nutrition and life support from the mother's blood to the baby

The effects of smoking on a mother's body:

- Nicotine tightens the blood vessels in the uterus, reducing blood flow to the placenta and baby
- Smoking produces carbon monoxide in the mother's blood stream, making less oxygen available to the placenta for baby
- The harmful chemicals contained within cigarettes are absorbed into the mother's blood stream and passed directly into the placenta and onto baby

Smoking cessation reduces the risk of:

- Miscarriage
- Low birth weight
- Pre-term or premature birth
- Poor lung development (leading to ongoing respiratory problems)
- Birth defects including cleft palate & heart defects
- Weak immune system
- Need for neonatal intensive care
- SIDS
- Future behavioural problems (including ADHD, substance use)
- Future tobacco smoking

When is the best time to stop?

- Stopping at any time during pregnancy is beneficial, although cessation early in the pregnancy will always provide the best start in life for baby.
- Stopping before 20 weeks will provide the best results for birthweight, although smoking cessation in the second half of pregnancy will also result in birthweight improvements (24)

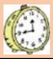	**Health changes after smoking cessation**
**After 30 minutes**	Blood pressure, heart rate, temperature of hands & feet become normal
**After 12 hours**	Carbon monoxide and oxygen levels in the blood return to normal
**After 48 hours**	Sense of taste and smell start to return to normal levels
**After 72 hours**	Bronchial tubes relax, and breathing is easier
**After 1 week**	Nicotine is flushed from your body
**After 2 weeks**	Circulation, breathing, and lung function improve
**After 1 month**	Coughing, sinus congestion and shortness of breath decrease

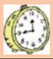	**Health changes after smoking cessation**
**After 2 years**	Risk of heart attack drops to that of a woman who has never smoked
**After 5 years**	Risk of stroke drops to normal; risk of lung cancer decreases by half
**After 10 years**	Risk of most types of cancer drops to normal
**After 20 years**	Risk of dying from smoking-related causes is similar to women who have never smoked

Preparing to stop smoking

**Tips to reduce tobacco**
➢ Use NRT; the slow release of nicotine from a patch will reduce the body's need for nicotine from cigarettes
➢ Get into the habit of only smoking outside the house and/or not in the car
➢ Try only smoking part of a cigarette and throwing the rest away
➢ Use the cigarette fading technique (below)
➢ Wherever possible, avoid those situations closely associated with smoking
➢ Take up new activities that distract and reduce available smoking time

**Cigarette Fading**	
1	Determine a number of cigarettes to smoke on Day 1. This should be at the low-end of the range smoked per day e.g. 10 if 10-15 is the average number of cigarettes smoked
2	Have only this number of cigarettes available. Hide all others e.g. give them to a non-smoking friend; put them in the freezer; have a neighbour hide them etc.
3	Take notice of the length of time in between each cigarette during the day.
4	If you achieve your smoking target, set a new, lower target for the following day. Make sure you gradually increase the amount of time between each cigarette
5	If a target is not achieved, try other strategies to reduce the number of cigarettes smoked
6	If still unable to meet the reduced target, stick with the original target number of cigarettes (e.g. 10), be mindful of your smoking and try implementing other strategies to reduce smoking.

Resisting the smoking urge

The DEEDS Strategy	
**Delay**	➢ Delay smoking for 15 minutes<br>➢ Delaying gets easier with practice, you can delay for longer periods of time<br>➢ Delaying means smoking less at the end of the day<br>➢ Strategies to delay:<br>– Delay first cigarette of the day e.g. until after breakfast<br>– Set smoke free hours e.g. no smoking from 9:00pm to 9:00am<br>– Put ashtrays and lighters is separate parts of the house or outside to make smoking less convenient
**Escape**	➢ Leave the situation that causes the craving if possible<br>➢ Not always so easy if you can't leave the children alone, especially if it is children that cause cravings. Try these strategies instead:<br>– Put a movie on for the children<br>– Take a mental vacation, imagine where you would like to be.<br>– Put on headphones and listen to music – you can see the children without hearing them<br>– Keep a treasure box of things from the Two Dollar Shop – if you need a break, a new toy could provide the distraction you require
**Evade**	➢ If possible, avoid places or situations where others will be smoking (when cravings to smoke ease, it will be easier to return to normal routine)<br>➢ Go places where smoking is not allowed e.g. movies, shopping centre, library<br>➢ Hang out with people who are non-smokers, or if not possible, chew gum and take a water bottle to<br>➢ Visit friends who have a non-smoking home
**Distract**	➢ Think of things to distract the mind from smoking, especially things that cannot be done while smoking:<br>– Play a game on your phone<br>– Wash dishes, do your hair, bath the dog<br>– Hammer nails<br>– Peel an orange<br>– Chew gum, seeds or something healthy<br>– Drink water, herbal tea or low carbonated/caffeinated drinks
**Substitute**	➢ When craving, substitute something that keeps hands & mouth busy<br>– Use NRT<br>– Chew NRT gum or regular gum<br>– Chew gum, seeds or something healthy<br>– Brush your teeth

Phase 3 - Supporting Smoking Cessation & Relapse Prevention

Identify high-risk situations & solutions

Looking back, looking forward

**Looking Back**
➢ Ask participant to think about what they have learnt about themselves in previous attempts to stop smoking (or other substances)<br>➢ Get them to identify the situations or events that occurred just prior to the lapse<br>➢ Identify any themes that emerge e.g. socialising with alcohol, with smoking friends<br>➢ Provide praise for good insight
**Looking Forward**
➢ Ask participant to recognise as many future high-risk situations as they can, based on these past experiences.<br>➢ Are there any new situations that are potential high-risk?<br>➢ Get participant to problem-solve possible solutions to apply to each of these situations.

Linking emotions to strategies

**Situation**	**Emotion / Feeling**	**Cigarette-free management**
➢ Mixing with friends / relatives / other who smoke cigarettes	➢ Left-out, isolated, alone	➢ Pre-prepare strategy before seeing them<br>➢ Use NRT<br>➢ Try mixing with others who don't smoke especially at the start
➢ After eating a meal	➢ Incomplete, nostalgic,	➢ Have a small, relatively healthy treat<br>➢ Use NRT

Strategies to deal with stress

Relationship between stress and nicotine levels

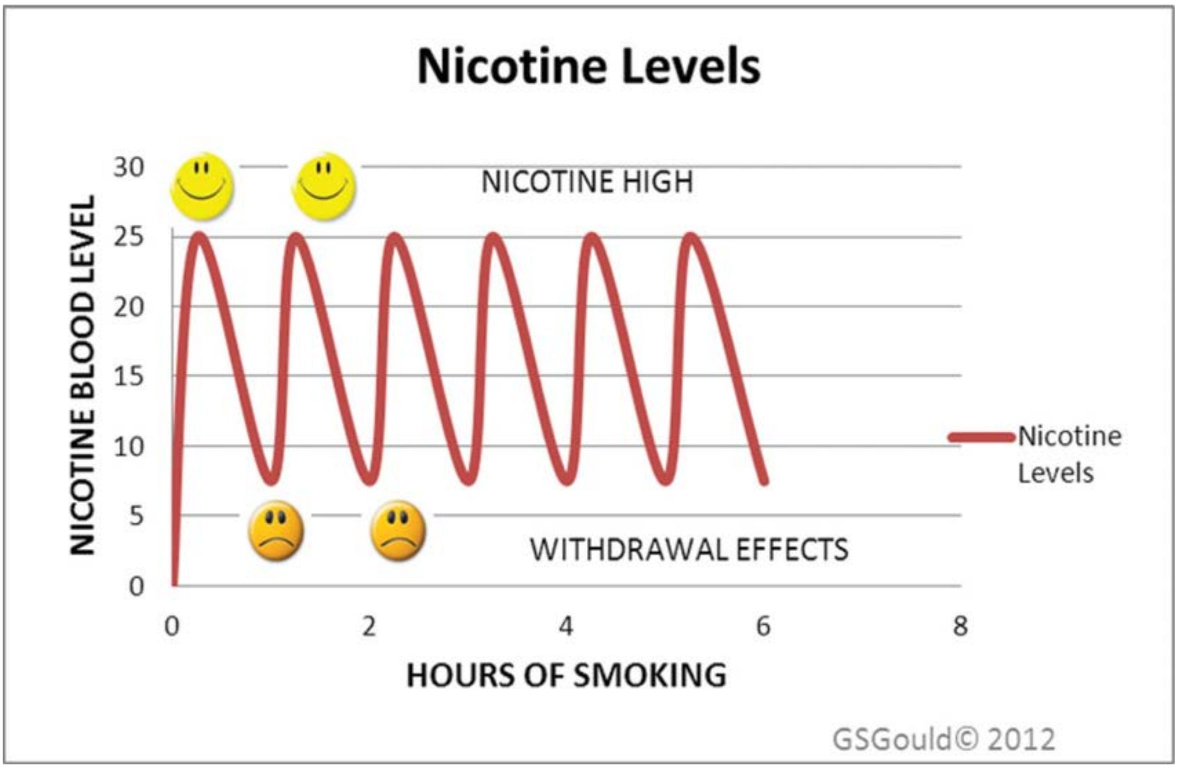

Mindfulness strategies

Breathing technique	
1	Find a comfortable quiet place to sit
2	Loosen any tight clothing
3	Close eyes and slow down breathing
4	Take slow, regular, deep breaths
5	Inhale through the nose to a long count of 5, hold for a count of 2, then exhale to a long count of 5
6	Focus on the sounds of your breathing and the feeling of your breath expanding your lungs and diaphragm
7	Continue for about 10 – 15 cycles

Creative Visualisation	
1	Find a comfortable quiet place to sit
2	Close eyes and slow down breathing
3	Imagine a picture, place or situation that makes you feel safe, relaxed and happy
4	Breathe in and out slowly and gently through the nose
5	Try and sense the smells, tastes and sounds of the target of your thoughts
6	Feel your body relax
7	Continue for at least 10 minutes

Urge Surfing	
1	Notice the urge. It may be a thought, a craving or a feeling.
2	Accept the feeling, knowing that it is a symptom of withdrawal and that it will subside
3	Sit and breathe through the urge, observing it without responding
4	Focus attention on those areas of the body most effected by the urge e.g. a knot in the stomach, tightness, itchiness.
5	After a short while, the craving will weaken and then completely pass by.

Self-compassion

Calming Self-talk	
➢	Remind yourself that making changes to tobacco use is difficult and it is normal to feel stressed. These feelings are part of nicotine withdrawal.
➢	Practice self-compassion and saying helpful things to yourself e.g. think about how a friend or family member might support or encourage you in a similar situation and then talk to yourself as they would
➢	Normalise what is happening to you. Almost everyone has similar difficulties when trying to stop smoking, you are not different, weak or stupid.
➢	Ignore or challenge the inner voice that criticises wrongdoings or bad decisions
➢	Practice being kind to yourself with kind affirmations or rewards for achievements, or reaching a set goal

Lapse and relapse in high risk situations

Pathway of relapse

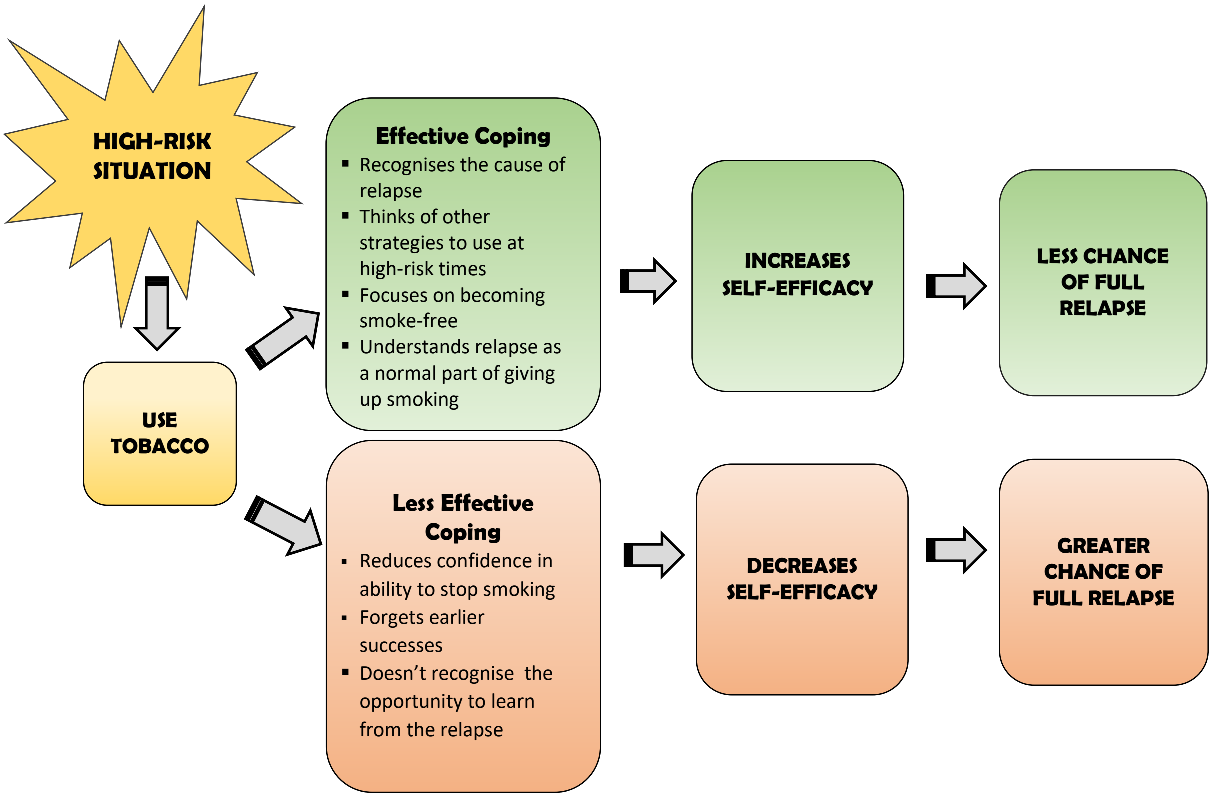

Dealing with cravings

**Coping with Cravings**
**Things to Do**
➢ The following list of physical things to do might be helpful:<br>- Use short-acting NRT to combat craving<br>- Switch tasks from the one being done as the craving hit<br>- Talk to a supportive friend or phone a Quitline<br>- Go for a quick walk or take the children to the park<br>- Take deep breaths<br>- Have a drink of water<br>- Chew gum, mints, fennel or anise seeds<br>- Clean your teeth<br>- Do relaxation exercises<br>- Take a shower<br>- Clean the house<br>- Colour in (either alone or with children)<br>- Do something with a non-smoker
**Things to Think**
➢ When it is too difficult to switch tasks, the following list of things to think can help:<br>- Consider the loss of hard-earned financial incentives from the study

Coping with Cravings
- Try mindfulness techniques (e.g. relaxation, urge surfing, visualisation, calming self-talk)<br>- Remember a new mantra 'I am a non-smoker'<br>- Attempt to figure out what bought on the craving for a cigarette<br>- Remember that the craving will be over soon<br>- Consider the positive changes that have already made and acknowledge them<br>- Think of protecting children and other house-hold members from second-hand smoke<br>- Think positive thoughts and remember the benefits of not smoking<br>- Don't think of a cigarette as a reward and think of other ways to reward oneself.

Problem solving techniques

Problem solving gives the opportunity to think about the difficulty, possible alternatives to solve the problem and the best solution. Ask the following questions when faced with a situation with multiple solutions:

Problem Solving Strategy	
1	**What is the problem?**<br>– *Define the problem clearly:*<br>– *E.g. household members are smoking inside and making it difficult to stop smoking*
2	**What is my goal?**<br>– *Determine the best possible outcome:*<br>– *E.g. to be smoke free and live happily with other household members*
3	**What are my options or alternatives?**<br>– *Think of as many options as possible:*<br>– *E.g. use more NRT to control cravings when others are smoking*<br>– *Use other strategies, such as DEEDS to resist smoking while others are*<br>– *Move out and find a different place to live*<br>– *Suggest that the house become smoke-free inside and set up a smoking area outside*<br>– *Make a designated smoke free area within the house e.g. the kitchen, bedroom etc.*<br>– *Spend more time away from home, with people who don't smoke, at least until smoking urges are under control*
4	**Weigh up these options**<br>– *Think about each alternative, weighing the good against the bad for each:*<br>– *e.g. NRT is relatively cheap and easy to use but tastes pretty bad; a smoke free home would be ideal, but housemates might not like to smoke outside; moving out would be expensive but could choose a smoke free home etc.*
5	**Select the most suitable option and DO IT!**

Conflict resolution

Tips to Avoid Conflict
➢ Use NRT to minimise withdrawal symptoms including irritability, mood swings etc.
➢ Prepare partner and/or those around you about the potential for intense emotions due to nicotine withdrawal and ask for their understanding.
➢ Avoid situations that have the potential to induce conflict
➢ Practice and use relaxation and grounding techniques
➢ Use distraction techniques e.g. DEEDS strategy
**Tips for Resolving Conflict**
➢ Compromise – be willing to bend and meet the other party half way
➢ Use humour – used respectfully, humour can diffuse heated situations. Focusing on oneself can be effective
➢ Apologise – people tend to lose the desire to remain in conflict once an apology has been issued.
➢ Share the responsibility – for both the conflict and its solution.
➢ Pick your battles – minor conflict is often not worth the effort required to resolve it.
➢ Get help – if conflicts cannot be resolved, mediation or advice from a neutral third party can be beneficial

Grounding technique:

Grounding technique
Find the following:
➢ **5** things you can **SEE**
➢ **4** things you can **HEAR**
➢ **3** things you can **TOUCH**
➢ **2** things you can **SMELL**
➢ **1** deep **BREATH**

Ongoing support

External quit smoking support providers

Quitlines
➢ **NSW Quitline** - 13 7848 (13 QUIT)<br>➢ **NSW Aboriginal Quitline** - 13 7848. ask to speak to the NSW Aboriginal Quitline<br>➢ **Multicultural Quitline**– see https://www.icanquit.com.au for times available<br>– Arabic - 1300 7848 03<br>– Vietnamese - 1300 7848 65<br>– Chinese - 1300 7848 36
**Smoking apps**
➢ **My Quit Buddy** – tips & distractions, tracking, progress graphs, facts,<br>➢ **Quit for you - Quit for two** – pregnancy focused, inspirational, fun exercises & games, personalised<br>➢ **Quittr** – includes fun games, personalised statistics, behavioural support<br>➢ **Stop Smoking - Mindfulness Meditation** – provides meditation/relaxation techniques

Promote long-term success:

Sleep Improvement

Sleep Hygiene
➢ Sleep hygiene is a term used to describe healthy sleeping habits.<br>➢ For the first few weeks of not smoking, it is common to have some trouble sleeping at night as your body goes through nicotine withdrawal.
**Tips for creating good sleep hygiene:**
➢ Avoid caffeine and any other chemicals that interrupt with sleep<br>➢ Go to bed at the same time each night to create a regular bedtime<br>➢ Create a dark, quiet and cool bedroom environment e.g. use sleep mask, earplugs<br>➢ Only use the bed for sleep & sex. Don't watch TV, eat or read from bed.<br>➢ Don't have sleeps during the day, or, have a short nap before 3:00pm (no more than 20 minutes)<br>➢ Have a warm bath before bed to help create sleepiness<br>➢ Don't watch the time during the night as this helps keep us alert and awake.<br>➢ Get some sunlight during the day to help create a healthy sleep-wake cycle<br>➢ If unable to sleep after 20 minutes, get up and do something relaxing until feeling tired enough to go to sleep

Sleep Hygiene
➢ If awake during the night & not able to go back to sleep, get up, keep the lights dim and do something non-stimulating such as listening to soft music or reading a telephone book for 20 minutes, then try again.<br>➢ Eat well during the day but avoid a heavy meal before bed. Have a glass of warm milk instead.<br>➢ Exercise during the day (preferably in the morning) as this helps to increase tiredness at night.

Strategies for a healthy lifestyle

A Healthy Lifestyle
➢ Weight gain can be a concern for many smokers, particularly women.<br>➢ The average weight gain after cessation is approx. 4-5 kg, although not everyone who stops smoking will gain weight (40).<br>➢ The health benefits of not smoking outweigh the risk of a few extra kilo's, so quitting is a good time to think about making changes to diet, exercise and other aspects of health.<br>➢ Once you see improvements in one area, it is easier to make changes in others. The benefits are far reaching and can include:<br>– Healthier pregnancy<br>– Improvements to health of other children<br>– Greater coping and improved mental health<br>– Better sleep<br>– Improved relationships<br>– Better sex<br>– More confidence<br>– Increases long-term happiness
**Diet**
➢ A healthy diet helps prevent weight gain<br>➢ Hunger is a withdrawal symptom, be prepared:<br>– snack on right foods e.g. fruit, nuts, carrots, yoghurt, boiled eggs, banana smoothie<br>– if possible, eat smaller meals more often<br>➢ Eat a good breakfast:<br>– morning cigarette cravings aren't as strong with a full stomach<br>– it improves mental alertness, concentration and mood, all things that are affected when withdrawing from cigarettes<br>➢ Food preparation is a distraction from smoking

A Healthy Lifestyle
**Exercise**
➢ Helps prevent weight gain by decreasing appetite<br>➢ Excellent for releasing brain endorphins that improve stress, anxiety and mood<br>➢ Great distraction from thoughts of smoking<br>➢ Eases withdrawal symptoms & cravings to smoke during and after exercising
**Relaxation**
➢ Great for relieving everyday stress particularly that associated with not smoking<br>➢ Great distraction from thoughts of smoking<br>➢ Try the following to relax:<br>– Deep breathing relaxation technique or creative visualisation listed in this manual<br>– Listening to music<br>– Squeezing a stress ball<br>– Practicing mindfulness / meditation<br>– Colouring in<br>– Walking

Reward Success

Rewards for success
➢ Simple, inexpensive, everyday rewards might include having a lie-in, listening to music, going for a walk, reading a book, watching a movie, eating some favourite food, or having a warm, relaxing bath.<br>➢ Calculate the potential amount of savings from not smoking between now and next session. Determine how this money could be used to enhance quality of life.<br>➢ Try putting the equivalent of a packet of cigarettes into a separate account each week. Have a savings goal e.g. a holiday or short escape, a trip to the theme park, buying new clothes after baby is born<br>➢ Eat out or order a nice take-away for a mid-week treat. The food will taste better and the break from cooking is appreciated more with a new baby or one on the way.<br>➢ See a movie, either alone, with a partner, or with children. It should be easier to sit through a movie without wanting a cigarette.<br>➢ Ask someone to look after children (if necessary) and book a massage.

Notes

____

____
